# African harps as units of cultural evolution: a cladistic analysis on their morphology

**DOI:** 10.1017/ehs.2025.10009

**Published:** 2025-07-29

**Authors:** Salomé Strauch, Guillaume Lecointre, Pierre Darlu, Sylvie Le Bomin

**Affiliations:** 1 UMR 7206 Eco-anthropologie, Muséum National d’Histoire Naturelle, Paris, France; 2 UMR 7205 Institut de Systématique, Evolution, Biodiversité, Muséum National d’Histoire Naturelle, Paris, France; 3 UMR 8223 Institut de Recherche en Musicologie, Sorbonne Université, Paris, France

**Keywords:** Harp, Africa, musical instrument evolution, cladistics, cultural evolution, phylogenetics

## Abstract

In Africa, harps exhibit significant morphological diversity, yet their historical trajectory remains largely underexplored. Phylogenetic reconstruction methods offer valuable tools for understanding this diversity and the relationships between groups of harps. This study is among the first to apply one of these methods, cladistics, to the morphology of a musical instrument, analysing 318 harps and 83 characters. We present a well-resolved phylogenetic tree, which shows several clades corresponding to geocultural regions, in alignment with ethnomusicological classifications. We show that this tree robustly represents the patterns of vertical transmission in the cultural evolution of harp morphology across Africa, despite the limited contribution of several tested characters. Additionally, a comparison with previous research reveals that characters coding decorations exert a minimal influence on the vertical evolution of these musical instruments. These findings provide valuable insights into the cultural evolution of harps on a continental scale, offering a clearer understanding of their diversity and revealing major evolutionary mechanisms.

## Graphical abstract

**Figure:**
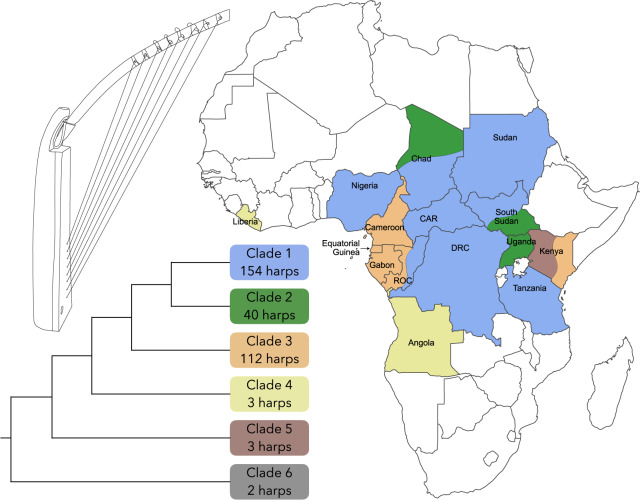

## 1. Introduction

The harp is a musical instrument characterized by its strings stretched between the soundbox and the neck, aligned perpendicular to the body of the instrument (Hornbostel and Sachs, 1961). Today, it is played on several continents, including Africa, from Mauritania to Uganda and in most Central African countries (DeVale, 2014; Leclair, 2007). The use of the harp on this continent for several millennia is evidenced by specimens found in Egypt, dated around 1500 BCE (Emerit et al., 2017), as well as by painted depictions discovered in Chad (Bailloud, 1997; Blench, 2013; Menardi Noguera, 2023), Algeria (Kubik, 2010), and Egypt (Emerit et al., 2017), dating from 1500 to 500 BCE. Africa is particularly notable for its rich diversity of harps, in terms of morphology (size, materials, shapes, decorations; Speranza, 1999), their contexts of use (entertainment, ritual ceremonies, court music; Arom, 2019; Bruguière, 1999), and the music they produce. Despite this, the history of the harp in Africa remains poorly understood, largely due to the preservation challenges of these instruments over time (Lawergren, 1981) and the lack of written records due to oral transmission in local populations (Arom, 1988; Shelemay, 2008; Vansina, 1980). This implies that in Central Africa, the earliest mentions of the instrument are linked to European travellers’ accounts (Fürniss, 1999; Praetorius, 1619). Furthermore, in the literature, African harps are more frequently studied as music-producing objects with cultural significance in specific contexts (Herndon, 1974; Johnson, 1995), rather than as material and cultural objects, resulting in fragmented descriptions (Strauch & Le Bomin, 2024).

Phylogenetic reconstruction methods, derived from life sciences, can help distinguish different groups of African harps and explain their diversity, potentially providing more insights into their history. These methods are increasingly applied to cultural materials, such as folktales (Tehrani, 2013), languages (Ben Hamed et al., 2005; Gaillard-Corvaglia et al., 2008; Gray & Jordan, 2000; Koile et al., 2022), lithic assemblages (Rineau et al., 2023), initiation rites (Bentley et al., 2021), myths (d’Huy, 2012, 2013), concepts (Charbonnat et al., 2014), manuscripts (Platnick & Cameron, 1977; Robinson, 2016; Schmidt et al., 2003), figures of the tree of life (Fisler et al., 2020), woven textiles (Buckley, 2012), and Buddha statues (Marwick, 2012). Although music is a shared cultural heritage among all past and present human populations, only a few phylogenetic analyses have been conducted in this field (Aguirre-Fernández et al., 2021; Le Bomin et al, 2016; Tëmkin & Eldredge, 2007; Windram et al., 2022).

Applying phylogenetic reconstruction methods to African harps is therefore particularly relevant, first because their history and diversity are underexplored, and second because, as musical instruments, they hold significant cultural value across various environments, lifestyles, and sociocultural practices of the populations that use them. Furthermore, the deep evolutionary history of musical instruments (including harps) in hunter-gatherer populations in Central Africa (Padilla-Iglesias et al., 2024) shows the interest for such phylogenetic analyses. One such method, cladistics, has previously been used to study the evolution of the morphology and decorations of 318 African harps (Strauch, 2023). The resulting phylogenetic tree showed three main groups of harps. These groups align with the geographical distributions documented in the literature and are predominantly represented by harps from Gabon, the Democratic Republic of Congo, and Uganda. The results also showed that the evolution of the morphology and decorations of African is not solely driven by vertical transmission processes (from one generation to the next), as there is a significant proportion of innovations that are transmitted horizontally (between peers).

Here, we distinguish between morphological characteristics, which deal with the organological and functional features of harps, and decorative characteristics, which have no impact on the music produced and are intended to decorate the harp (i.e. carvings, paintings, and engravings). In this article, we present a cladistic analysis focused solely on the morphology of these harps. It is therefore identical to previous analysis (Strauch, 2023), except for the exclusion of decorative characters. This enables a direct comparison between the two analyses and allows us to assess the impact of decorative features on the topology of the phylogenetic tree (i.e. by determining whether its structure changes and how these changes affect the relationships between harps). This approach also narrows the focus to the morphological evolution of the musical instruments. However, it is important to note that the reverse is not feasible, as not all harps are decorated, which would prevent a cladistic analysis focusing solely on decorative features for the same 318 harps. Our goals are therefore to (1) identify groups of harps and the morphological features that define them; (2) discuss the role of different transmission processes (vertical, horizontal) of innovations in the evolution of African harp morphology, and their sharing as a result of selective pressures or retroactive reappearances; and (3) assess the impact of removing decorative features on the resulting phylogenetic tree by comparing it to the previously obtained one.

## 2. Materials and methods

### 2.1. Cladistics: theoretical principles and parsimony method

Phylogenetic systematics, or cladistics, is an approach to systematics based on the kinship relationships between evolutionary units and on inherited modifications (Hennig, 1966). In this study, we employ the terms evolutionary unit (EU) (Meacham, 1984) and individual (Tëmkin & Eldredge, 2015), as they are applicable to both biological and cultural entities. An EU is a group of individuals (as a species), whereas an individual is a tangible representative of that EU (as a living organism).

Among cladistic methods, parsimony is the one employed in this study. Its goal is to produce phylogenetic trees and select the tree or trees that minimize the number of evolutionary changes. A cladogram (a phylogenetic tree produced by cladistic methods) is generated through the parsimony analysis of a EUs–characters matrix. This matrix is a two-dimensional table, with the EUs under study listed in rows and the characters listed in columns. A character has at least two states and corresponds to an attribute observed on all individuals of an EU that exhibits at least two observable forms (Darlu et al., 2019). For instance, if the character ‘Number of strings’ has the states ‘5 strings’, ‘6 strings’, and ‘8 strings’, it reflects a specific attribute of harps (they have strings), and the states represent observable forms (some harps have 5 strings, others have 6, and some have 8). However, defining a character and its states is not merely a description of an attribute and its forms; it formalizes the hypothesis that two forms observed in two EUs are similar enough to be derived from a single ancestral form (Barriel, 2011; Tassy, 2022). When defining a character and assigning different states to different EUs, the hypothesis is that individuals (and thus the EUs to which they belong) sharing a particular form of an attribute (e.g. harps with 5 strings) are more closely related to each other than to individuals (and the EUs they belong to) with a different form of that attribute (e.g. harps with 6 and 8 strings). The way a character is defined reflects the hypothesis being tested in the analysis. For instance, if it is hypothesized that 8-string harps evolved separately from 5-string and 6-string harps, coding the character ‘Number of Strings’ with three states to represent each observable form (‘5 strings’, ‘6 strings’, and ‘8 strings’) does not formalize this evolutionary hypothesis. In contrast, defining the character with the two states ‘fewer than 8 strings’ and ‘8 strings’ does. Thus, the definition of character states represents a primary homology (de Pinna, 1991): it is a hypothesis of homology, that is, of identity, which serves as an argument for determining the evolutionary relationships between EUs. The EUs–characters matrix therefore records all the states (in the cells) for each character (in the columns) exhibited by the EUs (in the rows) (Pleijel, 1995).

A cladogram is the result of a parsimony analysis conducted on the EUs–characters matrix. It groups together the studied EUs by maximizing the contiguity of identical character states (a principle of coherence; Rieppel, 2004, 2005). There are three consequences: it maximizes hierarchical congruence between the groupings of EUs supported by different character states; it minimizes the number of steps, that is, the number of character state changes (parsimony); and if the graph is read through the theory of evolution, attributes shared by EUs are inherited from a common ancestor.

On the cladogram, the EUs can belong to monophyletic groups, or clades, defined by one or more synapomorphies. A synapomorphy is a derived character state shared by all members of a clade; it represents the form of an attribute that originated in a single ancestral EU and was inherited by all its descendants (Hennig, 1966; Tassy & Fischer, 2021). It is a character state for which the hypothesis of homology has not been refuted, representing a secondary homology (de Pinna, 1991). Secondary homology refers to a relationship between two structures that are similar enough to be considered inherited from the same ancestral structure, that is, homologous structures (Bock, 1973; Brower & Schawaroch, 1996). The hypothesis of homology (i.e. potential homology or primary homology) is tested through cladistic analysis and is thus central to it (Cracraft, 1981). As Patterson (1982: 34) notes, ‘The idea that every worthwhile hypothesis of homology specifies a hierarchy of groups is all I wish to emphasize here. The force of a hypothesis of homology is that the inclusive group is monophyletic, by virtue of the homology.’

Homoplastic characters states are forms of attributes that are similar but not shared by an ancestral EU of all its descendants. The analysis has revealed these states to be different among these EUs, that is, that the hypothesis of homology is refuted.

According to cladistic theory (conceptualized within the life sciences), it is assumed that nature can be ‘ordered in a single specifiable pattern which can be represented by a branching diagram or hierarchical classification’ (Platnick, 1979: 538). Therefore, in cladistic methods, transmission can only be vertical, meaning that a given (non-homoplastic) character state is inherited from an ancestral EU by all its descendants.

However, it is recognized that transmission processes are as diverse for cultural EUs as they are for some biological units: innovations can be transmitted vertically (from an ancestor to its descendants), horizontally (between descendants of different ancestors), or obliquely (from an ancestor to the descendants of another ancestor) (Creanza et al., 2017; L. L. Cavalli-Sforza, 1986). For simplicity, both horizontal and oblique transmission processes will be referred to as horizontal transmission (Borgerhoff Mulder et al., 2006). This type of transmission cannot be unambiguously identified by cladistic methods, as they only consider vertical transmission and homoplasy can have different causes: horizontal transmission, but also reversion, similar selection pressure (e.g. availability of materials, musical styles), independent appearances, or character coding errors.

This implies that all character states transmitted horizontally will be considered homoplastic by the analysis, that is, as similar and not shared by an ancestral EU and all of its descendants. Conversely, one could also assume that homoplastic character states may be due to horizontal transfer. Nevertheless, applying these methods to cultural material allows for testing numerous hypotheses and determining hierarchical relationships between the considered EUs. These methods account for the diversity resulting from evolution, whether biological or cultural.

### 2.2. The 318 harps of the matrix

**Figure 1.**
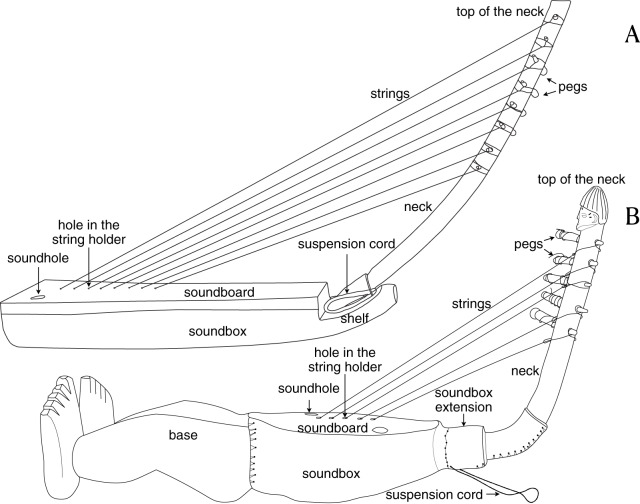
Illustration of two harps and their constituent parts. **(A)** Orungu harp described in Gabon by Sylvie Le Bomin, in the field. **(B)** Ngbaka harp from Democratic Republic of Congo described by Noé Coussot at the AfricaMuseum (Belgium).

In Africa, harps consistently feature a soundbox, a neck and strings, with the vast majority also including a soundboard, a string holder, and tuning pegs. Additionally, some harps have a soundbox extension, a shelf, and/or a base (Fig. 1). Across the continent, each of these structural components exhibits variation in size, shape, material, and ornamentation, which may include engraving, carving, and/or painting.

The cladistic analysis is conducted on the same 318 harps as those used by Strauch (2023), including 314 African harps for the ingroup and four non-African harps for the outgroup (Supplementary Table S1).

**Figure 2.**
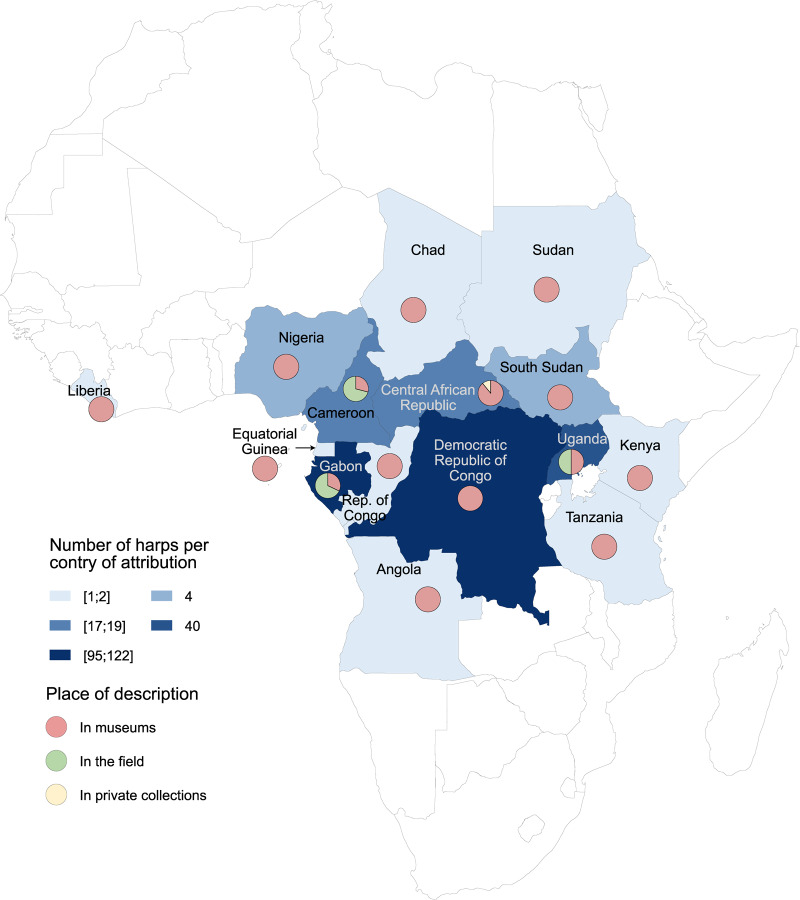
Geographic distribution of the 314 African harps in the matrix by country of attribution, showing the number of harps per country and the proportion described in museums, in the field, or in private collections.

The country and population of attribution are known for all harps on the ingroup: they come from 15 different countries and are attributed to 71 different populations. They were all described between 2016 and 2023. Among them, 68.5% were described in European and African museums, 30.9% in the field, and 0.6% in private collections (Fig. 2). Each harp is complete or is missing only a few pegs and/or strings. Those missing are considered identical to those present on the harp in the analysis.

The four non-African harps added to the matrix serve as the outgroup, that is, they will be used to root the produced cladogram (Barriel & Tassy, 1998; Maddison et al., 1984). Two are attributed to Afghanistan, one to Burma, and one to Russia. They were described in 2023 at the Pitt Rivers Museum (England) and the Scenkonstmuseet (Sweden).

### 2.3. The 83 characters of the matrix

The 83 characters used in this analysis (Supplementary Table S2) are derived from the matrix used by Strauch (2023). Characters coding for decorations (engravings, paintings, and carvings) were either removed or modified, so that the matrix now contains only morphological characters (Table 1). They were defined on the basis of a data set compiled between 2016 and 2023, which describes 700 harps using, in particular, 223 morphological and decorative parameters. All descriptive parameters were reviewed to produce this matrix, considering their relevance from an evolutionary point of view (i.e. do they convey, as they stand, a homology hypothesis?) to determine whether they could be directly converted into characters, whether they needed to be modified, or whether they were considered irrelevant (see Strauch, 2023: 107–115 for more details). In this study, 21.7% of characters are equivalent to the corresponding parameters, 39.8% are modified from the associated parameters, and 38.6% are created for the analysis.

**Table 1.** Number of characters associated with the different parts of the harp in both analyses

	Number of characters	Number of characters in the matrix used by Strauch (2023)		
Parts of the harp	used in this analysis	Used unmodified in this analysis	Modified and used in this analysis	Not included in this analysis
General morphology	10	8	2	3
Soundbox	7	7	0	1
Soundboard	27	26	1	2
Soundholes	11	11	0	0
String holder	6	6	0	0
Neck	6	6	0	2
Pegs	13	13	0	1
Strings	3	3	0	0
Carvings	0	0	0	29
Total	83	80	3	38

The three modified characters originally coded for the presence and engraving of the soundbox extension, the shelf, and the seal reinforcement, with three states: ‘absent’, ‘present without engraving’, and ‘present with engraving’. These characters were renamed ‘Presence of a soundbox extension’, ‘Presence of a shelf’, and ‘Presence of a seal reinforcement’ and now have the two states ‘present’ and ‘absent’.

Among the 38 excluded characters, three coded for the colours of the paintings, five for the presence of engraving on various harp parts, one for the presence of paint on the soundboard, and 29 for the carvings on the harp. As mentioned previously, removing decorative characters from the matrix allows for an evaluation of their impact on the topology of the resulting cladogram. It also significantly reduces the number of missing data in the matrix (coded as ‘?’), with the one used by Strauch (2023) containing 19% missing data compared to 9.60% in this analysis. This is because not all harps are carved and when one is, not all its constituent parts are carved. Such characters cannot be coded for a non-carved harp and are therefore inapplicable. They can lead to impossible state reconstructions for nodes in the cladogram (Platnick et al., 1991), even though some authors believe that it is irrelevant to exclude characters simply because of their missing data (Poe & Wiens, 2000).

Of the 83 characters in the matrix, seven characters code for continuous measurements (1–3: maximum overall length, height, and width of the harp; 12, 13: soundbox length and maximum width in the middle of the soundbox; 66, 67: neck straight length and neck curved length to straight length ratio). They were discretized by considering four classes of the same amplitude for each character, and therefore four states. Additional harp measurements were not taken into account in the analyses, as their discretization poses several problems in cladistics. For example, this procedure relies on many arbitrary choices, and the addition or removal of harps can potentially alter intervals and therefore character states (Stevens, 1991; Thiele, 1993).

Fifty-three characters are binary and 30 are multistate. One character is uninformative (9: Presence of metallic plates next to the string holder), that is, only one harp has the state ‘1: Presence’ and the other 317 have the state ‘0: Absence’. For each character, the attribution of a given state to a harp has been made based on its photographs and its description. With the exception of modified characters, no character states have been changed between the matrix used by Strauch (2023) and that of this analysis.

### 2.4. The parsimony analysis

The matrix includes 318 harps and 83 characters (Supplementary Table S3). The parsimony analysis was performed with PAUP* 4.0a169 (Swofford, 2003), with a heuristic search, using random stepwise addition with the TBR (Tree Bisection and Reconnection) branch-swapping algorithm. Both convergences and reversals were allowed, following Wagner parsimony. ACCTRAN optimization (‘accelerated transformation’), which favours reversals, was applied here (Darlu et al., 2019; Swofford & Maddison, 1992) because it maximizes the number of secondary homologies in the tree (de Pinna, 1991). The ‘max-length = 0’ rule was enforced, meaning that any unsupported branch in the tree, under all possible character optimizations, was collapsed (i.e. when its maximum length is 0). All characters were treated as unordered, meaning that any transformation from one state to another for each character ‘costs’ one step (Fitch, 1971). All traits were supposed to evolve independently and neither characters nor state transformations were weighted. The resulting trees were rooted using the four outgroups.

## 3. Results

**Figure 3.**
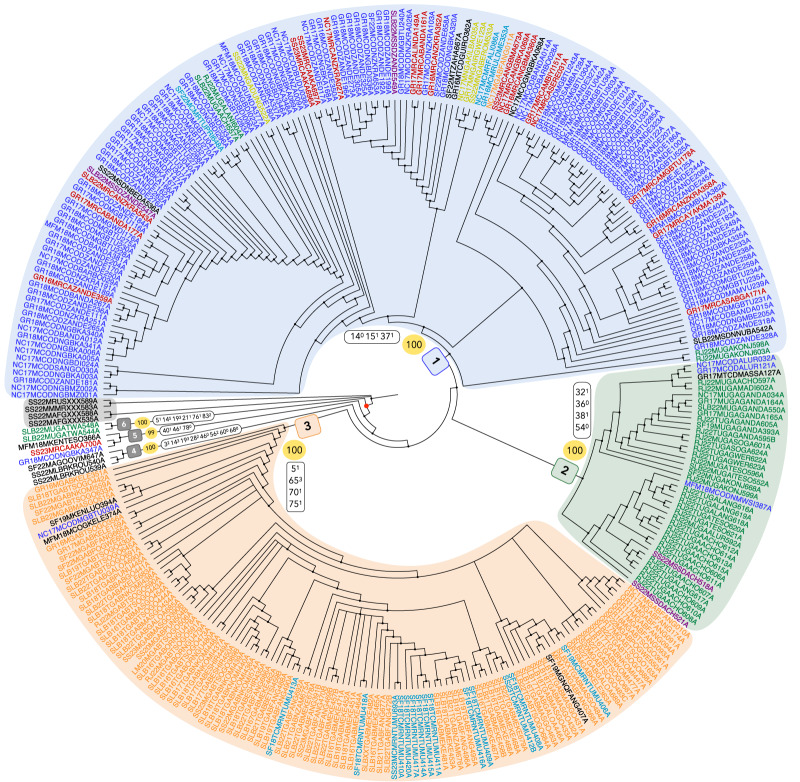
Cladogram of the majority-rule (50%) consensus tree (1581 steps), retained from 500,000 trees (1581 steps). Branch lengths are not informative. The cladogram is rooted with the four outgroup harps, circled in grey. The identifiers of the harps represented by at least four harps in the matrix are colour-coded according to their country of origin: Democratic Republic of the Congo (dark blue), Gabon (orange), Cameroon (light blue), Central African Republic (red), Uganda (green), Nigeria (dark yellow), and South Sudan (plum). The ingroup is marked by a red dot at its node. The six clades that are numbered in the figure (1–6) correspond to those that are examined in detail in this paper, with the first three colour-coded in blue, green, and orange, respectively, based on the main geographical attribution of the harps composing each clade. The numbers in the yellow circles correspond to the proportion of trees where the node is retained, while white boxes indicate the character state transformations at each node, with the state noted in superscript.

The parsimony analysis provided 500,000 most parsimonious trees of 1581 steps. They are summarized via majority-rule (50%) consensus tree of 1581 steps (Fig. 3). This type of consensus only retains clades present on more than 50% of the trees (Margush & McMorris, 1981). Consistency and retention indexes (CI and RI) are used as measures of homoplasy in the matrix related to the most parsimonious solutions and vary between 0 and 1. Considering 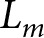 the minimum number of transformations if the characters are binary, 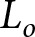 the length of the most parsimonious tree, and 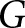 the number of steps if all changes occur along the terminal branches, they are defined as 
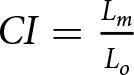
 (Kluge & Farris, 1969) and 
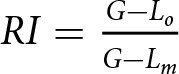
 (Archie, 1989; Farris, 1989).

Although these indices are calculated differently, they can be interpreted in a similar manner: ‘if the tree fits the data perfectly, with no extra steps, they take on the value of 1’ (Goloboff, 1991: 218), and their values decrease as homoplasy increases. For this consensus tree, CI = 0.092 and RI = 0.706.

The ingroup is monophyletic, comprising the 314 African harps analysed. This clade is characterized by a soundbox length between 12 and 36.625 cm (character 12, state 0: 12^0^), the absence of soundholes in the upper right (49^0^) and lower left (52^0^) parts of the soundboard, the presence of an internal string holder (56^1^), a neck plugged in the soundbox (65^0^), and twisted strings (82^1^). The transformations for characters 56 and 82 are ambiguous, meaning they could have occurred elsewhere in the tree without additional steps under a different optimization (Agnarsson & Miller, 2008).

Only two clades include all the harps from a single country, and only those harps: the two harps from Afghanistan are grouped together within the outgroup and the two harps from Liberia are more closely related to each other than to the harp from Angola in clade 4 (Fig. 3). No other clade meets both conditions of containing only harps from a given country and all the harps from that country. However, six clades can be distinguished, with the first three containing 97.45% of the harps in the ingroup. The vast majority (or at least half, depending on the country) of the harps from a given country can be assigned to one of these main clades (Table 2).

**Table 2.** Number of harps of the ingroup by country of attribution in each clade

Country of attribution	Number of harps	Distribution within clades						Main clade
		Clade 1	Clade 2	Clade 3	Clade 4	Clade 5	Clade 6	
DRC	122	95.9%	2,5%	0.8%		0.82%		1
Gabon	95	1.05%		98.95%				3
Uganda	40	10%	85%				5%	2
CAR	19	94.7%				5.3%		1
Cameroon	17	17.65%		82.35%				3
Nigeria	4	100%						1
South Sudan	4	50%	50%					1, 2
ROC	2	50%		50%				1, 3
Kenya	2			50%		50%		3, 5
Liberia	2				100%			4
Sudan	2	100%						1
Chad	2	50%	50%					1, 2
Angola	1				100%			4
EQG	1			100%				3
Tanzania	1	100%						1

The term ‘main clade’ is used here to refer to the clade containing the majority of harps attributed to a given country. Clade 1 is the main clade for the harps from the Democratic Republic of the Congo (DRC), the Central African Republic (CAR), Nigeria, Sudan, and Tanzania. Clade 2 is the main clade for the harps from Uganda, and clade 3 is the main clade for the harps from Gabon, Cameroon, and Equatorial Guinea (EQG). Clade 4 is the main clade for the harps from Liberia and Angola. The harps from the Republic of the Congo (ROC) are equally divided between clades 1 and 3, and the harps from Kenya are 50% in clade 3 and 50% in clade 5.

Clade 1 includes 154 harps (Fig. 3), characterized by a cylindrical soundbox (14^0^) with a domed bottom (15^1^) and tight lacing that attaches the soundboard to the soundbox (37^1^). The transformations for characters 14 and 37 are ambiguous.

Clade 2 includes 40 harps, characterized by the presence of nails on the front of the harp to attach the soundboard to the soundbox (32^1^), by the fact that the lacing that attaches the soundboard is loose (38^1^) and absent from the back of the harp (36^0^), and by the absence of a soundhole in the lower right of the soundboard (54^0^). Only the transformation for character 36 is unambiguous.

Clade 3 includes 112 harps, characterized by the presence of a shelf (5^1^), a neck resting on the soundbox (65^3^), pegs with a choked knob (70^1^), and the presence of a gap between the body and the head of the pegs (75^1^). None of the transformations are ambiguous.

Clade 4 includes three harps, characterized by a maximum overall width between 26.9 and 37.95 cm (3^2^), an ellipsoidal morphology of the soundbox (14^3^) and the soundboard (19^3^), wooden and metal nails (28^2^), absence of soundholes (46^0^), an external string holder (56^2^), strings tied to or around the string holder (60^0^), and the absence of pegs (68^0^). The transformations for characters 3, 14, 19, and 28 are ambiguous.

Clade 5 includes three harps, characterized by the presence of hairs on all the soundboard (40^1^), the presence of only one soundhole (46^1^), and pegs that do not increase in diameter between the body and the head (78^0^). The transformation for character 40 is ambiguous.

Clade 6 includes two harps, characterized by the presence of a shelf (5^1^), a bucket-shaped soundbox (14^5^), an ellipsoidal soundboard (19^3^) made of reptile skin (21^1^), notched pegs (76^1^), and the presence of five strings (83^2^). The transformations for characters 14 and 19 are ambiguous.

CI and RI can also be computed for individual characters (Supplementary Table S4). Only six characters have a CI of 1 (7.23% of all characters): they code for the presence of a sound-modifying element on the neck (7), presence of metallic plates next to the string holder (9), soundbox material (11), presence of mirlitons (44), presence of holes in the string holder (59), and presence of pegs (68). Six additional characters have an CI of 0.5 (7.23%): they code for the presence of a bone collar around the string holder (8), presence of a musical instrument attached to the harp (10), presence of a soundboard (18), dorsal position of the nails (33), soundholes location (45), and peg mounting angle (73). All other characters have a CI below 0.5 (85.54%): 41 of them have a CI less than or equal to 0.1 (49.40%) and four have a CI below 0.03. They code for the presence of a suspension cord (43), presence of a gap between the body and the head of the pegs (75), increased peg diameter (78), and presence of twists on the strings (82).

Characters with a CI of 1 also have an RI of 1 (6.02% of all characters), except for character 9 as it is uninformative. Fifty-three characters have an RI greater than or equal to 0.5 (63.85%) and 25 have an RI below 0.5 (30.12%), including eight characters with an RI equal to 0. They code for the presence of a bone collar around the string holder (8), presence of metallic plates next to the string holder (9), presence of a musical instrument attached to the harp (10), presence of seams to attach the soundboard (26), presence of ligatures to attach the soundboard (27), soundholes location (45), presence of a fork on the pegs (77), and presence of a ridge on the pegs (80).

## 4. Discussion

### 4.1. Main clades and geographic distribution of harps

Apart from the harps from Liberia, harps attributed to the same country within the ingroup do not form monophyletic groups on the cladogram (Fig. 3). However, they are predominantly grouped within the same main clade (Table 2). For instance, whereas 95.9% of harps from the DRC are found in the main clade 1, three harps attributed to this country are located in clade 2. Two of these are manufactured by the Alur people, who inhabit both the DRC and Uganda (Tamisier, 1998): this may explain the placement of these two DRC harps within clade 2, a clade primarily represented by Ugandan harps. The third harp is attributed to the Nyamwezi people, a population based in Tanzania (Tamisier, 1998) but not in the DRC. The geographical and ethnological information associated with the harp is therefore inconsistent, and at least one of them can be assumed to have been incorrectly recorded in museum collections (Gansemans, 1996). A similar situation is thought to have occurred with the only harp attributed to Gabon that falls outside clade 3. It is in clade 1 and exhibits morphological characteristics that are significantly different from the other Gabonese harps studied. Such attribution errors may explain the unexpected position in another clade of the affected harps.

It can also be explained by the geographical position of the attributed country and by where the attributed population live. For example, South Sudan is represented by four harps in the matrix: half are placed in clade 1 and are attributed to the Zande population, which is present in South Sudan, the DRC, and CAR. As shown by our results (Table 2), clade 1 is the main clade for the DRC and CAR harps. The other half of the South Sudanese harps are in clade 2 and are attributed to the Acholi population, which is found in South Sudan and Uganda (clade 2 being the main clade of Ugandan harps). Given South Sudan’s geographical position between the DRC, CAR (clade 1), and Uganda (clade 2), and the distribution of the Zande and Acholi populations across these countries, the division of South Sudanese harps between clades 1 and 2 is consistent. A similar pattern is observed for harps from the ROC, which are split between clades 1 and 3. Geographically, ROC is located between Gabon and the DRC. The ROC harp in clade 1 is attributed to the Ngbaka people, who also reside in the DRC and CAR, whereas the ROC harp in clade 3 is associated with the Kele people, a population also found in Gabon (clade 3 being the main clade for Gabonese harps).

Regarding the harps attributed to Cameroon, 82.35% are placed in clade 3 and 17.65% in clade 1. All Cameroonian harps from clade 3 are attributed to the Fang Ntumu population and come from the South region, near the border with Gabon and Equatorial Guinea (main clade 3). In contrast, the three Cameroonian harps from clade 1 are attributed to the North and Far North regions, bordering Nigeria and Chad (main clade 1). These harps are linked to three different populations: the two Fali and Uldeme harps are grouped together, whereas the Tupuri harp is positioned much further apart within clade 1 (Fig. 3). Thus, the results clearly demonstrate a division of Cameroonian harps between clades 1 and 3 based on their region of attribution, which aligns with the well-documented differences in harp morphology and usage between the northern and southern regions of the country (Fernando, 1999; Fürniss, 1999; Rivière, 1999).

Although the main clade of Ugandan harps is clade 2, four harps from this country are found in clade 1, likely due to the geographic proximity between Uganda and the DRC. Clade 6 consists of two Ugandan harps, which form a sister group to all other harps within the ingroup. These two harps are attributed to the Twa population, a collective term encompassing many Pygmy groups across the DRC, ROC, and Uganda (Bahuchet, 2012). Notably, these are the only harps in the matrix specifically made for tourists, which likely accounts for their markedly different morphological characteristics compared to other Ugandan harps and the rest of the ingroup.

**Figure 4.**
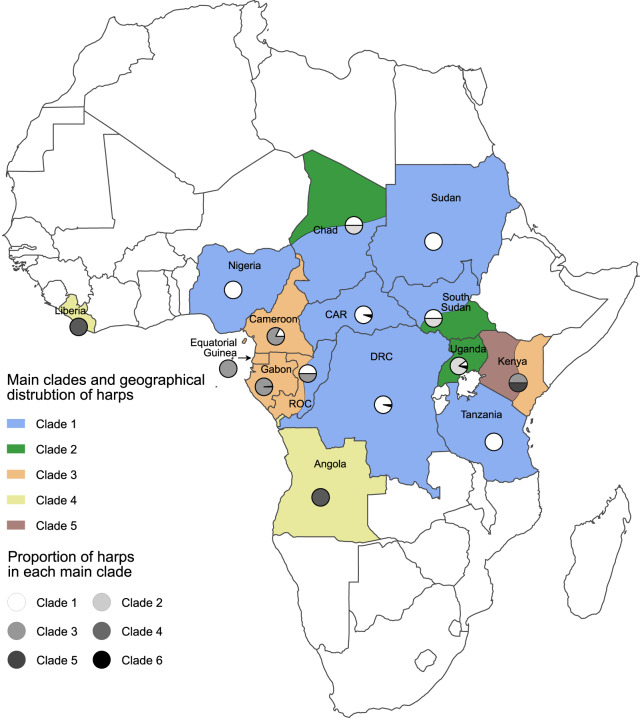
Geographical distribution of harps in the main clades defined on the cladogram of the majority-rule (50%) consensus. For countries with harps attributed equally to two main clades, their assignment to these clades is represented by two arbitrarily distributed colours.

Thus, although harps from the same country do not form a monophyletic group within the ingroup, except for those from Liberia, a clear geographical coherence is still observed in the cladogram (Fig. 4). Indeed, harps from the same geographic area are primarily grouped together, although there are exceptions that can be explained by, in particular, their manufacture (e.g. tourist harps), misidentification in museums, or their attribution data (countries, regions, and populations). Therefore, the use of their country of attribution as a distinguishing criterion has its limits, as the use of harps in Africa is largely determined by the populations who play them, and the distribution of these populations does not align with African administrative borders, which are recent and the result of colonialism, with little relevance at the local scale (Coquery-Vidrovitch, 2012). However, country attribution is more frequently known in museums, and above all more reliable, than ethnological designation (population, linguistic group) or more precise geographical identifications (region, town, etc.). Additionally, for some countries that are equally represented in two clades (South Sudan, ROC, Kenya, Chad), these results should be interpreted cautiously due to the small number of harps attributed to those countries (Table 2). It would be valuable to include more harps from each country to confirm their placement in one clade or another.

The first three main clades identified in this analysis confirm numerous geographic descriptions found in the literature, where three groups of harps are typically distinguished. Jadinon (2014) describes a continuous change in harp morphology along the axis connecting southern Cameroon, Gabon, EQG, DRC, and Uganda. Similarly, Wachsmann (1964) defined and mapped three groups of harps based on how the neck is attached to the soundbox, without, however, reference to countries or borders. Yet, these groups align closely with the composition of the first three main clades identified here.

As with country attributions, harps assigned to the same population do not form monophyletic groups and are more widely dispersed within a given main clade. This indicates that it is not possible to determine a harp’s population of origin based solely on its morphological features. Nevertheless, such attribution is often made in ethnomusicology (de Dampierre, 1991; Speranza, 1995), suggesting that ‘the information available in museums is incomplete and, unfortunately, often incorrect’ (Speranza, 1999: 74).

### 4.2. Characters, phylogenetic signal, and transmission of innovations

The application of cladistics to cultural data encounters a major issue, frequently discussed in the literature (Boyd et al., 1997; Cavalli-Sforza & Feldman, 1981; Greenhill et al., 2009; Guglielmino et al., 1995; Lukas et al., 2021; Steele et al., 2010; Tëmkin, 2016): cultural innovations can be transmitted either vertically (from an ancestor to all its descendants) or horizontally (between descendants of different ancestors or from one ancestor to the descendants of others). Vertical transmission of innovations produces homologies and implies a hierarchical pattern (nested sets of EUs from the most general attributes to the most particular attributes), which is represented by cladograms. Conversely, horizontal transmission leads to similarities that are not inherited, resulting in a non-hierarchical evolutionary pattern. Thus, horizontal transmission cannot be captured by cladistic methods, which were developed to investigate biological evolution (Hennig, 1966). Although cladistics can be useful when applied to cultural data, these methods may not be entirely suited to them. Specifically, cladistic methods are well adapted to biological evolution and to the vertical transmission of cultural traits (L. Cavalli-Sforza, 2005), whereas horizontal transmission of cultural traits can significantly impact the results obtained (Currie et al., 2010). Therefore, the application of cladistics in cultural evolution is only relevant when the innovations being studied are transmitted primarily or exclusively through vertical transmission. Consistency and retention indices help estimate the tree-likeness of the data (Bryant et al., 2005) and the reliability of the results – that is, to what extent the characters and their states, as encoded in the matrix, reflect a hierarchical structure and, thus, are homologous (and vertically transmitted). These indices are frequently used in cultural evolution studies to detect horizontal transmission (Collard et al., 2006; Fisler et al., 2020; Nunn et al., 2010).

The analysis showed that 7.23% of the characters have a CI of 1, whereas 85.54% have a CI below 0.5. Additionally, 36.14% of characters have an RI above 0.7, and 30.12% have an RI below 0.5. Therefore, while some characters are fully homologous (and thus vertically transmitted), the majority are not. Nevertheless, the cladogram is well resolved, exhibiting relatively few polytomies, and its nodes are recovered in nearly all 500,000 trees retrieved by the analysis. Moreover, our results show that the geographical distribution of the main clades on the cladogram is both consistent per se and aligned with descriptions from the literature (Fig. 4). Such a structured tree could not have been produced if the characters were random. This suggests that the hierarchical structure observed in the consensus tree, although weakly supported by the characters (as indicated by the low CI and RI values), lacks a viable alternative hypothesis based on the data used. On another argument line, one must keep in mind that there is no contradiction between low CIs and RIs and a well-resolved cladogram. This conjunction can be explained by the fact that slightly homoplastic characters (e.g. binary characters with two changes in the tree, then with a CI of 0.5) can provide structure in a large cladogram (Philippe et al., 1996) if changes of the homoplastic character state are well separated by several nodes. Moreover, it is noteworthy that these indices yield contradictory results in this study, both for numerous characters and the cladogram (CI = 0.092, RI = 0.706): the former indicates a high degree of homoplasy, whereas the latter suggests the opposite. Several biases are associated with the use of these indices (Strauch, 2023), and, as Archie highlights, ‘it is not apparent from the literature how CI and RI values should be used’ (Archie, 1996: 181).

Despite this, there is still a significant portion of our data set that does not contribute to the vertical evolution of harp morphology, which could be due to several factors: (1) the homology hypothesis is still assumed to be correct, but the way the character is coded does not adequately describe it; (2) the character may be transmitted horizontally, thus not following a hierarchical pattern; or (3) the character may not be evolutionarily relevant (e.g. if its transformations are random) and should be excluded from the cladistic analysis. It would therefore be beneficial to re-examine the entire set of characters and their transformations across the cladogram, identifying those that should be recoded or removed to reduce ‘noise’ in the data set and maximize the vertical fraction of the phylogenetic signal within the matrix.

For example, we can examine the 12 characters with a CI of 1 (indicating that the number of transformations from one state to another is minimal on the tree) or 0.5 (indicating that these characters require twice as many steps as theoretically necessary). Among them, five relate to features that alter the sound produced by the harp: buzzers (characters 7, 8, 9), mirlitons (character 44), and instruments attached to the harp (character 10). These characters, therefore, exhibit a strong phylogenetic signal (compared to other characters) both individually and collectively, as they all concern the modification of the harp’s natural sound. They can be retained for future analyses.

Similarly, the four characters with a CI below 0.03 are particularly homoplastic (and thus not vertically transmitted). Two of these characters involve tuning pegs (characters 75 and 78), and while they theoretically require only one transformation (as they have only two states), the cladogram shows 38 transformations for character 75 and 35 transformations for character 78. Therefore, these characters do not contribute to the vertical evolution of harp morphology. Moreover, the use of the harp and the music it produces are not constrained by the morphology of the pegs, as long as they are long enough to attach the strings and wide enough to fit into the neck. This suggests that the morphology of the pegs is relatively free, allowing for a high degree of shape variability, where selection may be more arbitrary and individual rather than codified. Therefore, we assume that these characters are not evolutionarily relevant and should be excluded from future analyses.

The seven characters coding for discretized continuous measurements (characters 1, 2, 3, 12, 13, 66, and 67) are also homoplastic. Their CI is consistently below 0.1, with each character showing 38–60 transformations on the cladogram. However, these characters provide information on the general dimensions of the harp, its soundbox, and its neck. These dimensions indirectly reflect the instrument’s potential use and context of application (e.g. can it be played while walking, or must it be placed on the ground?). Therefore, we suggest that the reason these characters do not contribute to the vertical evolution of harp morphology is that their coding does not capture potential homologies. In future analyses, it may be worthwhile to order the states of these characters (Farris, 1970) or to test their relevance with an alternative coding approach, by transforming them into qualitative variables or into quantitative continuous characters (Goloboff et al., 2006).

### 4.3. Morphology and decorations of African harps

In cladistics, a classical method for testing the ‘noise’ introduced by certain characters is to exclude them from the analysis. This has been done in the present study, which focuses solely on morphological characters, as the 41 characters coding for harp decorations included in Strauch’s analysis (Strauch, 2023) were removed or reformulated (Table 1). Strauch’s study produced a 50% majority-rule consensus of one million most parsimonious trees, with a length of 2400 steps. It was well-resolved, with the vast majority of its nodes consistently recovered in the one million trees. Its CI was 0.092, and its RI was 0.650. The CIs of the two analyses are therefore identical, but the RI for the morphology-only analysis is slightly higher (RI = 0.706).

The cladogram obtained in our study shows the same geographical consistency as the one presented by Strauch (2023: 120), with the main clades including harps from the same countries as in that study. Figure 4, inspired by the figure produced by Strauch (2023: 134), illustrates similar distributions. The only difference concerns the two harps from Liberia and the one from Angola: in this study, they are grouped together in clade 4 (Fig. 4), whereas they are included within the main clade of Gabonese harps in the analysis that incorporated decorative elements (Strauch, 2023).

In both studies, harps from the same countries are generally distributed similarly across the main clades. However, some differences can be observed. The proportion of harps from three countries (Cameroon, Gabon, Uganda) found in their respective main clades slightly decreases in this analysis compared to Strauch’s (2023) by 0.55%, 1.05%, and 2.50%, respectively. Conversely, this proportion increases notably for the CAR, the DRC, and Nigeria (5.24%, 13.10%, and 25%, respectively) in this analysis.

The results obtained by considering only the morphology of the harps are overall similar to those obtained when also taking their decorations (sculptures, paintings, engravings) into account, in terms of topology, indices (CI and RI), and geographical congruence of the cladogram. This indicates that the characters coding for decorations do not have a major impact on the vertical evolution of harps; in other words, they do not contribute significantly enough to induce noticeable changes in the cladogram. The only observed changes after removing these characters are generally positive, as the RI is higher, and the proportion of harps from the DRC, CAR, and Nigeria found in their main clade 1 is increased in this analysis. It is therefore interesting to note that decorations do not exert a significant impact on the evolution of harps compared to their morphology, despite the fact that they are often prioritized, particularly anthropomorphic sculptures, in various categorizations of harps (Strauch & Le Bomin, 2024). This study highlights the importance of examining the morphology of harps as a whole in their description and study, rather than focusing solely on their decorations.

## 5. Conclusion

This study is among the first to apply cladistic methods to the morphology of a musical instrument (Strauch, 2023; Tëmkin & Eldredge, 2007) and to examine such an extensive data set (318 harps, 83 characters). The consensus cladogram reveals several major clades, whose geographical distribution aligns with established ethnomusicological descriptions. These groups are primarily represented by harps from the DRC, Gabon, Uganda, and Liberia. However, the hierarchical structure of the cladogram is weakly supported by the characters, indicating that the majority of them, as coded in the matrix, do not significantly contribute to the vertical evolution of harp morphology. Additionally, the results were compared to those of Strauch (2023), showing that harp decorations have only a minor influence on vertical evolutionary patterns compared to morphological features.

This study highlights the relevance of cladistic methods when applied to cultural artefacts and has successfully characterized the evolution of harps on a continent-wide scale in Africa. In relation to the field of ethnomusicology, this research invalidates certain practices, such as attributing a harp to a specific population based solely on its morphological similarity to others, and prioritizing sculptures as key elements in the morphological descriptions of harps.

## Supporting information

Strauch et al. supplementary material Strauch et al. supplementary material

## Data Availability

The data used in this study can be found in the supplementary material and in a public repository on GitLab: https://gitlab.com/cultural-evolution/african-harps-as-units-of-cultural-evolution/-/tree/main.

## Supplementary material

The supplementary material for this article can be found at https://doi.org/10.1017/ehs.2025.10009.
