# Supplementary material for: African harps as units of cultural evolution: a cladistic analysis on their morphology
Source: Evol Hum Sci. 2025 Jul 29;7:e31. doi: 10.1017/ehs.2025.10009 (PMC12645324; doi:10.1017/ehs.2025.10009)
Supplement: Strauch et al. supplementary material [file S2513843X25100091sup001.pdf]

Supplementary Tables for
« African harps as units of cultural evolution: a cladistic analysis on their morphology »

Salomé Strauch[1,*], Guillaume Lecointre[2], Pierre Darlu[1], Sylvie Le Bomin[1,3]

1. Eco-anthropologie, UMR 7206 MNHN-CNRS-UPCité, Muséum National d'Histoire Naturelle, Paris, France.
2. Institut de Systématique, Evolution, Biodiversité, UMR 7205 CNRS-MNHN-SU-EPHE PSL, Muséum National d'Histoire Naturelle, Paris, France.
3. Institut de Recherche en Musicologie, UMR 8223 CNRS-SU-BNF-Min.Culture, Sorbonne Université, Paris, France.

* Corresponding author, email: salome.strauch@edu.mnhn.fr

Supplementary Table S1 - List of harps in the matrix

Harps 230, 245, 246, and 247 in this table are the outgroup harps; their rows are shaded in gray for easier identification.

Abbreviations:

AFG: Afghanistan

CAR: Central African Republic

DRC: Democratic Republic of Congo

EQG: Equatorial Guinea

ROC: Republic of the Congo

SSD: South Sudan

N°	Harp identifier in the matrix	Place of description	Described by (Year)	Attributed country	Attributed population
**1**	NC17MCODNGBMZ001A	AfricaMuseum, Belgium	N. Coussot (2017)	DRC	Ngbaka Manza
**2**	NC17MCODNGBMZ002A	AfricaMuseum, Belgium	N. Coussot (2017)	DRC	Ngbaka Manza
**3**	NC17MCODNGBKA003A	AfricaMuseum, Belgium	N. Coussot (2017)	DRC	Ngbaka

N°	Harp identifier in the matrix	Place of description	Described by (Year)	Attributed country	Attributed population
4	NC17MCODNGBKA005A	AfricaMuseum, Belgium	N. Coussot (2017)	DRC	Ngbaka
5	NC17MCODNGBKA006A	AfricaMuseum, Belgium	N. Coussot (2017)	DRC	Ngbaka
6	NC17MCODMANZA009A	AfricaMuseum, Belgium	N. Coussot (2017)	DRC	Manza
7	NC17MGABFANG010A	AfricaMuseum, Belgium	N. Coussot (2017)	Gabon	Fang
8	NC17MGABFANG011A	AfricaMuseum, Belgium	N. Coussot (2017)	Gabon	Fang
9	NC17MCODBANDA012A	AfricaMuseum, Belgium	N. Coussot (2017)	DRC	Banda
10	NC17MCODBANDA014A	AfricaMuseum, Belgium	N. Coussot (2017)	DRC	Banda
11	NC17MCODBANDA015A	AfricaMuseum, Belgium	N. Coussot (2017)	DRC	Banda
12	NC17MCODZANDE017A	AfricaMuseum, Belgium	N. Coussot (2017)	DRC	Zande
13	NC17MCODNGBDI023A	AfricaMuseum, Belgium	N. Coussot (2017)	DRC	Ngbandi
14	NC17MCODNGBDI024A	AfricaMuseum, Belgium	N. Coussot (2017)	DRC	Ngbandi
15	NC17MCODNZKRA026A	AfricaMuseum, Belgium	N. Coussot (2017)	DRC	Nzakara
16	NC17MRCANZKRA027A	AfricaMuseum, Belgium	N. Coussot (2017)	CAR	Nzakara
17	NC17MCODBARBO028A	AfricaMuseum, Belgium	N. Coussot (2017)	DRC	Barambo
18	NC17MCODBARBO029A	AfricaMuseum, Belgium	N. Coussot (2017)	DRC	Barambo
19	NC17MCODSANGO030A	AfricaMuseum, Belgium	N. Coussot (2017)	DRC	Sango
20	NC17MRCASERE031A	AfricaMuseum, Belgium	N. Coussot (2017)	CAR	Sere
21	NC17MCODALUR032A	AfricaMuseum, Belgium	N. Coussot (2017)	DRC	Alur
22	NC17MUGAGANDA034A	AfricaMuseum, Belgium	N. Coussot (2017)	Uganda	Ganda
23	NC17MNGAKILBA035A	AfricaMuseum, Belgium	N. Coussot (2017)	Nigeria	Kilba
24	NC17MCODBUDU038A	AfricaMuseum, Belgium	N. Coussot (2017)	DRC	Budu
25	NC17MCODMGBTU039A	AfricaMuseum, Belgium	N. Coussot (2017)	DRC	Mangbetu
26	NC17MCODMGBTU040A	AfricaMuseum, Belgium	N. Coussot (2017)	DRC	Mangbetu
27	NC17MCODMGBTU041A	AfricaMuseum, Belgium	N. Coussot (2017)	DRC	Mangbetu
28	NC17MCODMGBTU042A	AfricaMuseum, Belgium	N. Coussot (2017)	DRC	Mangbetu
29	NC17MCOGNGBKA068A	Musée du Quai Branly - Jacques Chirac, France	N. Coussot (2017)	ROC	Bobanga
30	NC17MRCANGBKA069A	Musée du Quai Branly - Jacques Chirac, France	N. Coussot (2017)	CAR	Bobanga
31	NC17MCMRFALI086A	Musée du Quai Branly - Jacques Chirac, France	N. Coussot (2017)	Cameroon	Fali
32	GR17MCODNGBKA089A	AfricaMuseum, Belgium	G. Robic (2017)	DRC	Ngbaka
33	GR17MGABFANG091A	AfricaMuseum, Belgium	G. Robic (2017)	Gabon	Fang
34	GR17MGABFANG093A	AfricaMuseum, Belgium	G. Robic (2017)	Gabon	Fang
35	GR17MGABFANG094A	AfricaMuseum, Belgium	G. Robic (2017)	Gabon	Fang
36	GR17MCODNGBDI096A	AfricaMuseum, Belgium	G. Robic (2017)	DRC	Ngbandi

N°	Harp identifier in the matrix	Place of description	Described by (Year)	Attributed country	Attributed population
**37**	GR17MCODZANDE100A	AfricaMuseum, Belgium	G. Robic (2017)	DRC	Zande
**38**	GR17MCODZANDE101A	AfricaMuseum, Belgium	G. Robic (2017)	DRC	Zande
**39**	GR17MCODNZKRA103A	AfricaMuseum, Belgium	G. Robic (2017)	DRC	Nzakara
**40**	GR17MCODZANDE111A	AfricaMuseum, Belgium	G. Robic (2017)	DRC	Zande
**41**	GR17MCODMGBTU116A	AfricaMuseum, Belgium	G. Robic (2017)	DRC	Mangbetu
**42**	GR17MCODALUR121A	AfricaMuseum, Belgium	G. Robic (2017)	DRC	Alur
**43**	GR17MCODBARI122A	AfricaMuseum, Belgium	G. Robic (2017)	DRC	Bari
**44**	GR17MNGAIRGWE123A	AfricaMuseum, Belgium	G. Robic (2017)	Nigeria	Irigwe
**45**	GR17MCODMEJE125A	AfricaMuseum, Belgium	G. Robic (2017)	DRC	Meje
**46**	GR17MTCDMASSA127A	AfricaMuseum, Belgium	G. Robic (2017)	Chad	Massa
**47**	GR17MCODSAMBA128A	AfricaMuseum, Belgium	G. Robic (2017)	DRC	Samba
**48**	GR17MRCAYAKMA139A	Musée du Quai Branly - Jacques Chirac, France	G. Robic (2017)	CAR	Yakoma
**49**	GR17MCODZANDE147A	Musée du Quai Branly - Jacques Chirac, France	G. Robic (2017)	DRC	Zande
**50**	GR17MRCALINDA149A	Musée du Quai Branly - Jacques Chirac, France	G. Robic (2017)	CAR	Banda Linda
**51**	GR17MRCAMBIYI151A	Musée du Quai Branly - Jacques Chirac, France	G. Robic (2017)	CAR	Banda M’biyi
**52**	GR17MGABKELE159A	Musée du Quai Branly - Jacques Chirac, France	G. Robic (2017)	Gabon	Kélé
**53**	GR17MRCABANDA161A	Musée du Quai Branly - Jacques Chirac, France	G. Robic (2017)	CAR	Banda
**54**	GR17MCODZANDE162A	Musée du Quai Branly - Jacques Chirac, France	G. Robic (2017)	DRC	Zande
**55**	GR17MGABTSOGO163A	Musée du Quai Branly - Jacques Chirac, France	G. Robic (2017)	Gabon	Tsogho
**56**	GR17MUGAGANDA164A	Musée du Quai Branly - Jacques Chirac, France	G. Robic (2017)	Uganda	Ganda
**57**	GR17MUGAGANDA165A	Musée du Quai Branly - Jacques Chirac, France	G. Robic (2017)	Uganda	Ganda
**58**	GR17MCODNGBKA166A	Musée du Quai Branly - Jacques Chirac, France	G. Robic (2017)	DRC	Ngbaka
**59**	GR17MCODNGBKA167A	Musée du Quai Branly - Jacques Chirac, France	G. Robic (2017)	DRC	Ngbaka
**60**	GR17MCODNGBKA168A	Musée du Quai Branly - Jacques Chirac, France	G. Robic (2017)	DRC	Ngbaka
**61**	GR17MCODZANDE170A	Musée du Quai Branly - Jacques Chirac, France	G. Robic (2017)	DRC	Zande

N°	Harp identifier in the matrix	Place of description	Described by (Year)	Attributed country	Attributed population
**62**	GR17MRCASABGA171A	Musée du Quai Branly - Jacques Chirac, France	G. Robic (2017)	CAR	Sabanga
**63**	GR17MGABFANG174A	Musée du Quai Branly - Jacques Chirac, France	G. Robic (2017)	Gabon	Fang
**64**	GR17MCODMGWDI176A	Musée du Quai Branly - Jacques Chirac, France	G. Robic (2017)	DRC	Mogwandi
**65**	GR17MRCABANDA177A	Musée du Quai Branly - Jacques Chirac, France	G. Robic (2017)	CAR	Banda
**66**	GR17MRCAMGBTU178A	Musée du Quai Branly - Jacques Chirac, France	G. Robic (2017)	CAR	Mangbetu
**67**	GR18MCODZANDE180A	AfricaMuseum, Belgium	G. Robic (2018)	DRC	Zande
**68**	GR18MCODZANDE181A	AfricaMuseum, Belgium	G. Robic (2018)	DRC	Zande
**69**	GR18MCODZANDE183A	AfricaMuseum, Belgium	G. Robic (2018)	DRC	Zande
**70**	GR18MCODZANDE184A	AfricaMuseum, Belgium	G. Robic (2018)	DRC	Zande
**71**	GR18MCODZANDE186A	AfricaMuseum, Belgium	G. Robic (2018)	DRC	Zande
**72**	GR18MCODZANDE189A	AfricaMuseum, Belgium	G. Robic (2018)	DRC	Zande
**73**	GR18MCODNZKRA191A	AfricaMuseum, Belgium	G. Robic (2018)	DRC	Nzakara
**74**	GR18MCODZANDE192A	AfricaMuseum, Belgium	G. Robic (2018)	DRC	Zande
**75**	GR18MCODZANDE196A	AfricaMuseum, Belgium	G. Robic (2018)	DRC	Zande
**76**	GR18MCODZANDE199A	AfricaMuseum, Belgium	G. Robic (2018)	DRC	Zande
**77**	GR18MCODZANDE204A	AfricaMuseum, Belgium	G. Robic (2018)	DRC	Zande
**78**	GR18MCODNGMBE205A	AfricaMuseum, Belgium	G. Robic (2018)	DRC	Ngombe
**79**	GR18MCODZANDE207A	AfricaMuseum, Belgium	G. Robic (2018)	DRC	Zande
**80**	GR18MCODZANDE208A	AfricaMuseum, Belgium	G. Robic (2018)	DRC	Zande
**81**	GR18MCODMGBTU227A	AfricaMuseum, Belgium	G. Robic (2018)	DRC	Mangbetu
**82**	GR18MCODMGBTU228A	AfricaMuseum, Belgium	G. Robic (2018)	DRC	Mangbetu
**83**	GR18MCODMGBTU230A	AfricaMuseum, Belgium	G. Robic (2018)	DRC	Mangbetu
**84**	GR18MCODMGBTU231A	AfricaMuseum, Belgium	G. Robic (2018)	DRC	Mangbetu
**85**	GR18MCODZANDE233A	AfricaMuseum, Belgium	G. Robic (2018)	DRC	Zande
**86**	GR18MCODMGBTU234A	AfricaMuseum, Belgium	G. Robic (2018)	DRC	Mangbetu
**87**	GR18MCODMGBTU235A	AfricaMuseum, Belgium	G. Robic (2018)	DRC	Mangbetu
**88**	GR18MCODZANDE236A	AfricaMuseum, Belgium	G. Robic (2018)	DRC	Zande
**89**	GR18MCODZANDE237A	AfricaMuseum, Belgium	G. Robic (2018)	DRC	Zande
**90**	GR18MCODZANDE238A	AfricaMuseum, Belgium	G. Robic (2018)	DRC	Zande
**91**	GR18MCODMAMVU239A	AfricaMuseum, Belgium	G. Robic (2018)	DRC	Mamvu
**92**	GR18MCODMGBTU240A	AfricaMuseum, Belgium	G. Robic (2018)	DRC	Mangbetu

N°	Harp identifier in the matrix	Place of description	Described by (Year)	Attributed country	Attributed population
**93**	GR18MCODMGBTU243A	AfricaMuseum, Belgium	G. Robic (2018)	DRC	Mangbetu
**94**	GR18MCODZANDE244A	AfricaMuseum, Belgium	G. Robic (2018)	DRC	Zande
**95**	GR18MCODZANDE245A	AfricaMuseum, Belgium	G. Robic (2018)	DRC	Zande
**96**	GR18MCODZANDE249A	AfricaMuseum, Belgium	G. Robic (2018)	DRC	Zande
**97**	GR18MCODZANDE250A	AfricaMuseum, Belgium	G. Robic (2018)	DRC	Zande
**98**	GR18MCODNZKRA251A	AfricaMuseum, Belgium	G. Robic (2018)	DRC	Nzakara
**99**	GR18MCODZANDE254A	AfricaMuseum, Belgium	G. Robic (2018)	DRC	Zande
**100**	GR18MCODZANDE258A	AfricaMuseum, Belgium	G. Robic (2018)	DRC	Zande
**101**	GR18MCODZANDE259A	AfricaMuseum, Belgium	G. Robic (2018)	DRC	Zande
**102**	GR18MCODMGBTU260A	AfricaMuseum, Belgium	G. Robic (2018)	DRC	Mangbetu
**103**	GR18MCODMGBTU261A	AfricaMuseum, Belgium	G. Robic (2018)	DRC	Mangbetu
**104**	GR18MCODZANDE262A	AfricaMuseum, Belgium	G. Robic (2018)	DRC	Zande
**105**	GR18MCODZANDE265A	AfricaMuseum, Belgium	G. Robic (2018)	DRC	Zande
**106**	GR18MCODBANDA267A	AfricaMuseum, Belgium	G. Robic (2018)	DRC	Banda
**107**	GR18MCODZANDE268A	AfricaMuseum, Belgium	G. Robic (2018)	DRC	Zande
**108**	GR18MCODMGBTU270A	AfricaMuseum, Belgium	G. Robic (2018)	DRC	Mangbetu
**109**	GR18MCODZANDE273A	AfricaMuseum, Belgium	G. Robic (2018)	DRC	Zande
**110**	GR18MCODMGBTU275A	AfricaMuseum, Belgium	G. Robic (2018)	DRC	Mangbetu
**111**	GR18MCODZANDE276A	AfricaMuseum, Belgium	G. Robic (2018)	DRC	Zande
**112**	GR18MCODMGBTU283A	AfricaMuseum, Belgium	G. Robic (2018)	DRC	Mangbetu
**113**	GR18MCODMGBTU285A	AfricaMuseum, Belgium	G. Robic (2018)	DRC	Mangbetu
**114**	GR18MXXXNGBDI296A	AfricaMuseum, Belgium	G. Robic (2018)	DRC	Ngbandi
**115**	GR18MCODMGBTU303A	AfricaMuseum, Belgium	G. Robic (2018)	DRC	Mangbetu
**116**	GR18MCODMGBTU304A	AfricaMuseum, Belgium	G. Robic (2018)	DRC	Mangbetu
**117**	GR18MXXXZANDE305A	AfricaMuseum, Belgium	G. Robic (2018)	DRC	Zande
**118**	GR18MCODMGBTU307A	AfricaMuseum, Belgium	G. Robic (2018)	DRC	Mangbetu
**119**	GR18MCODMGBTU311A	AfricaMuseum, Belgium	G. Robic (2018)	DRC	Mangbetu
**120**	GR18MCODZANDE318A	AfricaMuseum, Belgium	G. Robic (2018)	DRC	Zande
**121**	GR18MCODNGBKA320A	AfricaMuseum, Belgium	G. Robic (2018)	DRC	Ngbaka
**122**	GR18MCODZANDE321A	AfricaMuseum, Belgium	G. Robic (2018)	DRC	Zande
**123**	GR18MCODMGBTU322A	AfricaMuseum, Belgium	G. Robic (2018)	DRC	Mangbetu
**124**	GR18MCODZANDE323A	AfricaMuseum, Belgium	G. Robic (2018)	DRC	Zande
**125**	GR18MCODMGBTU324A	AfricaMuseum, Belgium	G. Robic (2018)	DRC	Mangbetu
**126**	GR18MCODZANDE325A	AfricaMuseum, Belgium	G. Robic (2018)	DRC	Zande

N°	Harp identifier in the matrix	Place of description	Described by (Year)	Attributed country	Attributed population
**127**	GR18MCODZANDE327A	AfricaMuseum, Belgium	G. Robic (2018)	DRC	Zande
**128**	GR18MCODZANDE328A	AfricaMuseum, Belgium	G. Robic (2018)	DRC	Zande
**129**	GR18MCODMGBTU331A	AfricaMuseum, Belgium	G. Robic (2018)	DRC	Mangbetu
**130**	GR18MCODZANDE334A	AfricaMuseum, Belgium	G. Robic (2018)	DRC	Zande
**131**	GR18MCODNGBKA335A	AfricaMuseum, Belgium	G. Robic (2018)	DRC	Ngbaka
**132**	GR18MCODNGBDI336A	AfricaMuseum, Belgium	G. Robic (2018)	DRC	Ngbandi
**133**	GR18MCODNGBKA337A	AfricaMuseum, Belgium	G. Robic (2018)	DRC	Ngbaka
**134**	GR18MCODNGBKA340A	AfricaMuseum, Belgium	G. Robic (2018)	DRC	Ngbaka
**135**	GR18MCODNGBKA341A	AfricaMuseum, Belgium	G. Robic (2018)	DRC	Ngbaka
**136**	GR18MCODNGBDI346A	AfricaMuseum, Belgium	G. Robic (2018)	DRC	Ngbandi
**137**	GR18MCODNGBKA347A	AfricaMuseum, Belgium	G. Robic (2018)	DRC	Ngbaka
**138**	GR18MCODNGBKA348A	AfricaMuseum, Belgium	G. Robic (2018)	DRC	Ngbaka
**139**	GR16MCODZANDE350A	Cité de la Musique, France	G. Robic (2016)	DRC	Zande
**140**	GR16MGABKELE351A	Cité de la Musique, France	G. Robic (2016)	Gabon	Kélé
**141**	GR16MRCANZKRA352A	Cité de la Musique, France	G. Robic (2016)	CAR	Nzakara
**142**	GR16CMRULDME353A	Cité de la Musique, France	G. Robic (2016)	Cameroon	Uldeme
**143**	GR16MGABTSOGO356A	Cité de la Musique, France	G. Robic (2016)	Gabon	Tsogho
**144**	GR16MRCANZKRA358A	Cité de la Musique, France	G. Robic (2016)	CAR	Nzakara
**145**	GR16MRCAZANDE359A	Cité de la Musique, France	G. Robic (2016)	CAR	Zande
**146**	GR16MRCANGBMA360A	Cité de la Musique, France	G. Robic (2016)	CAR	Ngbaka-Ma’bo
**147**	GR16MGABTSOGO361A	Cité de la Musique, France	G. Robic (2016)	Gabon	Tsogho
**148**	GR16MTCDGUIRO362A	Cité de la Musique, France	G. Robic (2016)	Chad	Gula-Iro
**149**	GR16MGABKELE363A	Cité de la Musique, France	G. Robic (2016)	Gabon	Kélé
**150**	MFM18MCODMGBTU364A	AfricaMuseum, Belgium	M.-F. Mifune (2018)	DRC	Mangbetu
**151**	MFM18MKENTESO366A	Musical Instrument Museum, Belgium	M.-F. Mifune (2018)	Kenya	Teso
**152**	MFM18MGABFANG368A	Musical Instrument Museum, Belgium	M.-F. Mifune (2018)	Gabon	Fang

N°	Harp identifier in the matrix	Place of description	Described by (Year)	Attributed country	Attributed population
**153**	MFM18MCOGKELE374A	Musical Instrument Museum, Belgium	M.-F. Mifune (2018)	ROC	Kélé
**154**	MFM18MCODGUNDA377 A	Musical Instrument Museum, Belgium	M.-F. Mifune (2018)	DRC	Gunda
**155**	MFM18MCODBANDA381 A	Musical Instrument Museum, Belgium	M.-F. Mifune (2018)	DRC	Banda
**156**	MFM18MCODMBUJA382 A	Musical Instrument Museum, Belgium	M.-F. Mifune (2018)	DRC	Mbuja
**157**	MFM18MCODNMWSI387 A	Musical Instrument Museum, Belgium	M.-F. Mifune (2018)	DRC	Nyamwesi
**158**	SF19MUGAGANDA393A	Ethnological Museum of Berlin, Germany	S. Fürniss (2019)	Uganda	Ganda
**159**	SF19MKENLUO394A	Ethnological Museum of Berlin, Germany	S. Fürniss (2019)	Kenya	Luo
**160**	SF19MCODZANDE404A	Ethnological Museum of Berlin, Germany	S. Fürniss (2019)	DRC	Zande
**161**	SF19MCMRNTUMU406A	Ethnological Museum of Berlin, Germany	S. Fürniss (2019)	Cameroon	Fang Ntumu
**162**	SF19MGNQFANG407A	Ethnological Museum of Berlin, Germany	S. Fürniss (2019)	EQG	Fang
**163**	SF18TCMRNTUMU408A	On the field, Cameroon	S. Fürniss (2018)	Cameroon	Fang Ntumu
**164**	SF18TCMRNTUMU409A	On the field, Cameroon	S. Fürniss (2018)	Cameroon	Fang Ntumu
**165**	SF18TCMRNTUMU410A	On the field, Cameroon	S. Fürniss (2018)	Cameroon	Fang Ntumu
**166**	SF18TCMRNTUMU411A	On the field, Cameroon	S. Fürniss (2018)	Cameroon	Fang Ntumu
**167**	SF18TCMRNTUMU413A	On the field, Cameroon	S. Fürniss (2018)	Cameroon	Fang Ntumu
**168**	SF18TCMRNTUMU414A	On the field, Cameroon	S. Fürniss (2018)	Cameroon	Fang Ntumu
**169**	SF18TCMRNTUMU415A	On the field, Cameroon	S. Fürniss (2018)	Cameroon	Fang Ntumu
**170**	SF18TCMRNTUMU416A	On the field, Cameroon	S. Fürniss (2018)	Cameroon	Fang Ntumu
**171**	SF18TCMRNTUMU417A	On the field, Cameroon	S. Fürniss (2018)	Cameroon	Fang Ntumu
**172**	SF18TCMRNTUMU418A	On the field, Cameroon	S. Fürniss (2018)	Cameroon	Fang Ntumu
**173**	SF18TCMRNTUMU420A	On the field, Cameroon	S. Fürniss (2018)	Cameroon	Fang Ntumu
**174**	SLB19TGABTSOGO430A	On the field, Gabon	S. Le Bomin (2019)	Gabon	Tsogho

N°	Harp identifier in the matrix	Place of description	Described by (Year)	Attributed country	Attributed population
**175**	SLB18TGABMASGO431A	On the field, Gabon	S. Le Bomin (2018)	Gabon	Massango
**176**	SLB18TGABMASGO432A	On the field, Gabon	S. Le Bomin (2018)	Gabon	Massango
**177**	SLB18TGABMASGO433A	On the field, Gabon	S. Le Bomin (2018)	Gabon	Massango
**178**	SLB18TGABMASGO434A	On the field, Gabon	S. Le Bomin (2018)	Gabon	Massango
**179**	SLB18TGABESHIR435A	On the field, Gabon	S. Le Bomin (2018)	Gabon	Eshira
**180**	SLB18TGABNKOMI436A	On the field, Gabon	S. Le Bomin (2018)	Gabon	Nkomi
**181**	SLB18TGABNKOMI437A	On the field, Gabon	S. Le Bomin (2018)	Gabon	Nkomi
**182**	SLB18TGABNKOMI438A	On the field, Gabon	S. Le Bomin (2018)	Gabon	Nkomi
**183**	SLB18TGABNKOMI439A	On the field, Gabon	S. Le Bomin (2018)	Gabon	Nkomi
**184**	SLB18TGABKNOMI440A	On the field, Gabon	S. Le Bomin (2018)	Gabon	Nkomi
**185**	SLB18TGABBABGO441A	On the field, Gabon	S. Le Bomin (2018)	Gabon	Babongo
**186**	SLB18TGABTSOGO442A	On the field, Gabon	S. Le Bomin (2018)	Gabon	Tsogho
**187**	SLB18TGABTSOGO443A	On the field, Gabon	S. Le Bomin (2018)	Gabon	Tsogho
**188**	SLB18TGABTSOGO444A	On the field, Gabon	S. Le Bomin (2018)	Gabon	Tsogho
**189**	SLB18TGABPUNU445A	On the field, Gabon	S. Le Bomin (2018)	Gabon	Punu
**190**	SLB17TGABGALOA446A	On the field, Gabon	S. Le Bomin (2017)	Gabon	Galoa
**191**	SLB17TGABNKOMI447A	On the field, Gabon	S. Le Bomin (2017)	Gabon	Nkomi
**192**	SLB17TGABNKOMI448A	On the field, Gabon	S. Le Bomin (2017)	Gabon	Nkomi
**193**	SLB17TGABESHIR449A	On the field, Gabon	S. Le Bomin (2017)	Gabon	Eshira
**194**	SLB17TGABESHIR450A	On the field, Gabon	S. Le Bomin (2017)	Gabon	Eshira
**195**	SLB18TGABMEKE451A	On the field, Gabon	S. Le Bomin (2018)	Gabon	Fang Mekè
**196**	SLB18TGABMEKE452A	On the field, Gabon	S. Le Bomin (2018)	Gabon	Fang Mekè
**197**	SLB18TGABMEKE453A	On the field, Gabon	S. Le Bomin (2018)	Gabon	Fang Mekè
**198**	SLB16TGABMASGO454A	On the field, Gabon	S. Le Bomin (2016)	Gabon	Massango
**199**	SLB167GABMEKE462A	On the field, Gabon	S. Le Bomin (2016)	Gabon	Fang Mekè
**200**	SLB16TGABMASGO463A	On the field, Gabon	S. Le Bomin (2016)	Gabon	Massango
**201**	SLB16TGABMEKE464A	On the field, Gabon	S. Le Bomin (2016)	Gabon	Fang Mekè
**202**	SLB16TGABMEKE465A	On the field, Gabon	S. Le Bomin (2016)	Gabon	Fang Mekè
**203**	SLB16TGABMEKE466A	On the field, Gabon	S. Le Bomin (2016)	Gabon	Fang Mekè
**204**	SLB16TGABMEKE467A	On the field, Gabon	S. Le Bomin (2016)	Gabon	Fang Mekè
**205**	SLB16TGABMEKE468A	On the field, Gabon	S. Le Bomin (2016)	Gabon	Fang Mekè
**206**	SLB16TGABMEKE471A	On the field, Gabon	S. Le Bomin (2016)	Gabon	Fang Mekè
**207**	SLB16TGABTSOGO473A	On the field, Gabon	S. Le Bomin (2016)	Gabon	Tsogho
**208**	SLB16TGABNKOMI477A	On the field, Gabon	S. Le Bomin (2016)	Gabon	Nkomi

N°	Harp identifier in the matrix	Place of description	Described by (Year)	Attributed country	Attributed population
**209**	SLB16TGABORNGU478A	On the field, Gabon	S. Le Bomin (2016)	Gabon	Orungu
**210**	SLB16TGABORNGU479A	On the field, Gabon	S. Le Bomin (2016)	Gabon	Orungu
**211**	SLB16TGABORNGU480A	On the field, Gabon	S. Le Bomin (2016)	Gabon	Orungu
**212**	SLB16TGABMEKE481A	On the field, Gabon	S. Le Bomin (2016)	Gabon	Fang Mekè
**213**	SLBXXTGABMEKE482A	?	S. Le Bomin (?)	Gabon	Fang Mekè
**214**	SLBXXTGABMASGO484A	?	S. Le Bomin (2018)	Gabon	Massango
**215**	SLB19TGABFANG488A	On the field, Gabon	S. Le Bomin (2019)	Gabon	Fang
**216**	SLB19TGABFANG490A	On the field, Gabon	S. Le Bomin (2019)	Gabon	Fang
**217**	SLB19TGABFANG495A	On the field, Gabon	S. Le Bomin (2019)	Gabon	Fang
**218**	SLB19TGABFANG496A	On the field, Gabon	S. Le Bomin (2019)	Gabon	Fang
**219**	LM20MGABTSOGO506A	Musée de l'Homme, Paris, France	L. Mariot (2020)	Gabon	Tsogho
**220**	SS20MGABTSOGO483C	Musée de l'Homme, France	S. Strauch (2020)	Gabon	Nkomi
**221**	SS20MGABPUN504B	Musée de l'Homme, France	S. Strauch (2020)	Gabon	Punu
**222**	SLB22MGABMASGO515A	Musée d'Angoulême, France	S. Le Bomin (2022)	Gabon	Massango
**223**	SS22MSSDACH518A	Pitt Rivers Museum, England	S. Strauch (2022)	SSD	Acholi
**224**	SS22MSSDACH521A	Pitt Rivers Museum, England	S. Strauch (2022)	SSD	Acholi
**225**	SS22MNGADUNG522A	Pitt Rivers Museum, England	S. Strauch (2022)	Nigeria	Jukun
**226**	SS22MCODMGBTU525A	Pitt Rivers Museum, England	S. Strauch (2022)	DRC	Mangbetu
**227**	SS22MNGABEROM533A	Pitt Rivers Museum, England	S. Strauch (2022)	Nigeria	Berom
**228**	SS22MAFGXXX535A	Pitt Rivers Museum, England	S. Strauch (2022)	AFG	?
**229**	SS22MSDNBEDA536A	Pitt Rivers Museum, England	S. Strauch (2022)	Sudan	Bedawi
**230**	SS22MLBRKROU539A	Pitt Rivers Museum, England	S. Strauch (2022)	Liberia	Krou
**231**	SS22MLBRKROU540A	Pitt Rivers Museum, England	S. Strauch (2022)	Liberia	Krou
**232**	SLB22MSDNNUBA542A	Pitt Rivers Museum, England	S. Le Bomin (2022)	Sudan	Nuba
**233**	SLB22MRCANZKRA543A	Pitt Rivers Museum, England	S. Le Bomin (2022)	CAR	Nzakara

N°	Harp identifier in the matrix	Place of description	Described by (Year)	Attributed country	Attributed population
**234**	SLB22MUGATWA544A	Pitt Rivers Museum, England	S. Le Bomin (2022)	Uganda	Twa
**235**	SLB22MSSDZANDE547A	Pitt Rivers Museum, England	S. Le Bomin (2022)	SSD	Zande
**236**	SLB22MUGATWA548A	Pitt Rivers Museum, England	S. Le Bomin (2022)	Uganda	Twa
**237**	SLB22MSSDZANDE549A	Pitt Rivers Museum, England	S. Le Bomin (2022)	SSD	Zande
**238**	SLB22MUGAGANDA550A	Pitt Rivers Museum, England	S. Le Bomin (2022)	Uganda	Ganda
**239**	SLB22MUGAITESO552A	Pitt Rivers Museum, England	S. Le Bomin (2022)	Uganda	Iteso
**240**	SLB22MUGAACH557A	Pitt Rivers Museum, England	S. Le Bomin (2022)	Uganda	Acholi
**241**	SLB22MGABNKOMI559A	Pitt Rivers Museum, England	S. Le Bomin (2022)	Gabon	Nkomi
**242**	SS22MGABNKOMI574A	Musical Instrument Museum, Belgium	S. Strauch (2022)	Gabon	Nkomi
**243**	SS22MMMRXXX583A	Scenkonstmuseet, Sweden	S. Strauch (2022)	Burma	?
**244**	SS22MAFGXXX588A	Scenkonstmuseet, Sweden	S. Strauch (2022)	AFG	?
**245**	SS22MRUSXXX589A	Scenkonstmuseet, Sweden	S. Strauch (2022)	Russia	?
**246**	RJ22MGABTSOGO590A	AfricaMuseum, Belgium	R. Jadinon (2022)	Gabon	Tsogho
**247**	RJ22MGABTSOGO591A	AfricaMuseum, Belgium	R. Jadinon (2022)	Gabon	Tsogho
**248**	RJ22MUGAALUR592A	AfricaMuseum, Belgium	R. Jadinon (2022)	Uganda	Alur
**249**	RJ22MUGATESO596A	Uganda National Museum, Uganda	R. Jadinon (2022)	Uganda	Teso
**250**	RJ22MUGAACHO597A	Uganda National Museum, Uganda	R. Jadinon (2022)	Uganda	Acholi
**251**	RJ22MUGAKONJ598A	Uganda National Museum, Uganda	R. Jadinon (2022)	Uganda	Konjo
**252**	RJ22MUGAKONJ599A	Uganda National Museum, Uganda	R. Jadinon (2022)	Uganda	Konjo
**253**	RJ22MUGASOGA601A	Uganda National Museum, Uganda	R. Jadinon (2022)	Uganda	Soga
**254**	RJ22MUGAMADI602A	Uganda National Museum, Uganda	R. Jadinon (2022)	Uganda	Madi
**255**	RJ22MUGAKONJ603A	Uganda National Museum, Uganda	R. Jadinon (2022)	Uganda	Konjo

N°	Harp identifier in the matrix	Place of description	Described by (Year)	Attributed country	Attributed population
**256**	RJ22MUGALANI604A	Uganda National Museum, Uganda	R. Jadinon (2022)	Uganda	Lani
**257**	RJ22TUGAGANDA605A	On the field, Uganda	R. Jadinon (2022)	Uganda	Ganda
**258**	RJ22TUGAACHO606A	On the field, Uganda	R. Jadinon (2022)	Uganda	Acholi
**259**	RJ22TUGAACHO607A	On the field, Uganda	R. Jadinon (2022)	Uganda	Acholi
**260**	RJ22TUGAACHO608A	On the field, Uganda	R. Jadinon (2022)	Uganda	Acholi
**261**	RJ22TUGAACHO609A	On the field, Uganda	R. Jadinon (2022)	Uganda	Acholi
**262**	RJ22TUGAACHO610A	On the field, Uganda	R. Jadinon (2022)	Uganda	Acholi
**263**	RJ22TUGAACHO611A	On the field, Uganda	R. Jadinon (2022)	Uganda	Acholi
**264**	RJ22TUGAACHO612A	On the field, Uganda	R. Jadinon (2022)	Uganda	Acholi
**265**	RJ22TUGAACHO613A	On the field, Uganda	R. Jadinon (2022)	Uganda	Acholi
**266**	RJ22TUGAACHO614A	On the field, Uganda	R. Jadinon (2022)	Uganda	Acholi
**267**	RJ22TUGAACHO615A	On the field, Uganda	R. Jadinon (2022)	Uganda	Acholi
**268**	RJ22TUGALANG616A	On the field, Uganda	R. Jadinon (2022)	Uganda	Lango
**269**	RJ22TUGALANG617A	On the field, Uganda	R. Jadinon (2022)	Uganda	Lango
**270**	RJ22TUGALANG618A	On the field, Uganda	R. Jadinon (2022)	Uganda	Lango
**271**	RJ22TUGALANG619A	On the field, Uganda	R. Jadinon (2022)	Uganda	Lango
**272**	RJ22TUGATESO620A	On the field, Uganda	R. Jadinon (2022)	Uganda	Teso
**273**	RJ22TUGATESO621A	On the field, Uganda	R. Jadinon (2022)	Uganda	Teso
**274**	RJ22TUGAGWER622A	On the field, Uganda	R. Jadinon (2022)	Uganda	Gwere
**275**	RJ22TUGAGWER623A	On the field, Uganda	R. Jadinon (2022)	Uganda	Gwere
**276**	RJ22TUGASOGA624A	On the field, Uganda	R. Jadinon (2022)	Uganda	Soga
**277**	RJ22TUGAGANDA595B	Uganda National Museum, Uganda	R. Jadinon (2022)	Uganda	Ganda
**278**	SF22MGABTSOGO633A	Musée national des Arts, Rites et Traditions du Gabon, Gabon	S. Fürniss (2022)	Gabon	Tsogho
**279**	SF22MGABTSOGO645A	Musée national des Arts, Rites et Traditions du Gabon, Gabon	S. Fürniss (2022)	Gabon	Tsogho
**280**	SF22MAGOOVIM647A	Overseas Museum, Germany	S. Fürniss (2022)	Angola	Ovimbund u
**281**	SF22MCMRTUPRI648A	Overseas Museum, Germany	S. Fürniss (2022)	Cameroon	Toupouri
**282**	SF22MGABPOGWE650A	Overseas Museum, Germany	S. Fürniss (2022)	Gabon	M'Pongwé
**283**	SF22MGABPOGWE651A	Overseas Museum, Germany	S. Fürniss (2022)	Gabon	M'Pongwé

N°	Harp identifier in the matrix	Place of description	Described by (Year)	Attributed country	Attributed population
**284**	SF22MCODNZKRA653A	Overseas Museum, Germany	S. Fürniss (2022)	DRC	Nzakara
**285**	SF22MCODZANDE658A	Overseas Museum, Germany	S. Fürniss (2022)	DRC	Zande
**286**	SF22MGABFANG665A	Völkerkundesammlung, Germany	S. Fürniss (2022)	Gabon	Fang
**287**	SF22MTZAHA667A	Völkerkundesammlung, Germany	S. Fürniss (2022)	Tanzanie	Ha
**288**	SF22MUGAKONJ668A	Völkerkundesammlung, Germany	S. Fürniss (2022)	Uganda	Konjo
**289**	SS23MRCANGBMA673A	Private collection, France	S. Strauch (2023)	CAR	Ngbaka-Ma’bo
**290**	SLB21TGABNZAM675A	On the field, Gabon	S. Le Bomin (2021)	Gabon	Fang Nzamane
**291**	SLB21TGABNZAM676A	On the field, Gabon	S. Le Bomin (2021)	Gabon	Fang Nzamane
**292**	SLB21TGABMEKE458B	On the field, Gabon	S. Le Bomin (2021)	Gabon	Fang Mekè
**293**	SLB21TGABBETSI459B	On the field, Gabon	S. Le Bomin (2021)	Gabon	Fang Betsi
**294**	SLB21TGABFANG677A	On the field, Gabon	S. Le Bomin (2021)	Gabon	Fang
**295**	SLB22TGABMEKE678A	On the field, Gabon	S. Le Bomin (2022)	Gabon	Fang Mekè
**296**	SLB22TGABMEKE679A	On the field, Gabon	S. Le Bomin (2022)	Gabon	Fang Mekè
**297**	SLB22TGABMEKE457B	On the field, Gabon	S. Le Bomin (2022)	Gabon	Fang Mekè
**298**	SLB22TGABAKELE683A	On the field, Gabon	S. Le Bomin (2022)	Gabon	Kélé
**299**	SLB22TGABTSOGO684A	On the field, Gabon	S. Le Bomin (2022)	Gabon	Simba, Tsogho
**300**	SLB22TGABGALOA685A	On the field, Gabon	S. Le Bomin (2022)	Gabon	Galoa
**301**	SLB22TGABFANG686A	On the field, Gabon	S. Le Bomin (2022)	Gabon	Fang
**302**	SLB22TGABTSOGO687A	On the field, Gabon	S. Le Bomin (2022)	Gabon	Tsogho
**303**	SLB22TGABTSOGO688A	On the field, Gabon	S. Le Bomin (2022)	Gabon	Tsogho
**304**	SLB22TGABGALOA689A	On the field, Gabon	S. Le Bomin (2022)	Gabon	Galoa
**305**	SLB22TGABGALOA690A	On the field, Gabon	S. Le Bomin (2022)	Gabon	Galoa
**306**	SLB22TGABTSOGO691A	On the field, Gabon	S. Le Bomin (2022)	Gabon	Tsogho
**307**	SLB22TGABFANG692A	On the field, Gabon	S. Le Bomin (2022)	Gabon	Fang
**308**	SLB22TGABFANG469B	On the field, Gabon	S. Le Bomin (2022)	Gabon	Fang
**309**	SLB22TGABFANG470B	On the field, Gabon	S. Le Bomin (2022)	Gabon	Fang
**310**	SLB22TGABFANG472B	On the field, Gabon	S. Le Bomin (2022)	Gabon	Fang
**311**	SS23MCMRNTUMU693A	Musée de l’Homme, France	S. Strauch (2023)	Cameroon	Fang Ntumu

N°	Harp identifier in the matrix	Place of description	Described by (Year)	Attributed country	Attributed population
**312**	SS23MGABMASGO390B	Sorbonne Université, France	S. Strauch (2023)	Gabon	Massango
**313**	SS23MGABFANG391B	Sorbonne Université, France	S. Strauch (2023)	Gabon	Fang
**314**	SS23MGABTSOGO389B	Sorbonne Université, France	S. Strauch (2023)	Gabon	Tsogho
**315**	SS23MRCAAKA696A	Musée de l'Homme, France	S. Strauch (2023)	CAR	Aka
**316**	SS23MRCAAKA697A	Musée de l'Homme, France	S. Strauch (2023)	CAR	Aka
**317**	SS23MRCAAKA700A	Private collection, France	S. Strauch (2023)	CAR	Aka
**318**	SS23TCMRNTUMU412B	Musée de l'Homme, France	S. Strauch (2023)	Cameroon	Fang Ntumu

Supplementary Table S2 - Characters used in the matrix

The symbol * in the first column indicates that the character has been modified from the matrix used by Strauch (2023).

N°	Character	States	
**1**	Maximum overall length of the harp (in centimeters)	0: [27; 64[<br>2: [101; 138[	1: [64; 101[<br>3: [138; 175]
**2**	Maximum overall height of the harp (in centimeters)	0: [15.3; 39.225[<br>2: [63.15; 87.075[	1: [39.225; 63.15[<br>3: [87.075; 111]
**3**	Maximum overall width of the harp (in centimeters)	0: [4.8; 15.85[<br>2: [26.9; 37.95[	1: [15.85; 26.9[<br>3: [37.95; 49]
**4***	Presence of a soundbox extension	0: Absence	1: Presence
**5***	Presence of a shelf	0: Absence	1: Presence
**6**	Presence of a base	0: Absence	1: Presence
**7**	Presence of a sound-modifying element on the neck	0: Absence	1: Presence
**8**	Presence of a bone collar around the string holder	0: Absence	1: Presence
**9**	Presence of metallic plates next to the string holder	0: Absence	1: Presence
**10**	Presence of a musical instrument attached to the harp	0: Absence	1: Presence

N°	Character	States
**11**	Soundbox material	0: Wood 1: Calabash<br>2: Tortoise shell 3: Plastic
**12**	Soundbox length (in centimeters)	0: [12; 36.625[1: [36.625; 61.25[<br>2: [61.25; 85.875[3: [85.875; 110.5]
**13**	Maximum width in the middle of the soundbox (in centimeters)	0: [2.6; 12.7[1: [12.7; 22.8[<br>2: [22.8; 32.9[3: [32.9; 43]
**14**	Soundbox morphology	0: Cylindrical 1: Naviform<br>2: Parallelepiped 3: Ellipsoidal<br>4: Truncated cylinder 5: Bucket-shaped
**15**	Shape of the bottom of the soundbox	0: Flat 1: Domed
**16**	Basal opening of the soundbox	0: Closed soundbox 1: Open soundbox
**17**	Presence of ridges on the soundbox	0: Absence 1: Presence
**18**	Presence of a soundboard	0: Absence 1: Presence
**19**	Soundboard morphology	0: Triangular pointing downwards<br>1: Triangular pointing upwards<br>2: Rectangular 3: Ellipsoidal<br>4: Indented
**20**	Soundboard material	0: Skin 1: Wood<br>2: Bark 3: Metal<br>4: Synthetic 5: Skin and metal
**21**	Taxonomic classification of the animal from which the skin originates	0: Mammal 1: Reptile<br>2: Mammal and reptile
**22**	Layout of the soundboard on the soundbox	0: Covers the sides of the soundbox<br>1: Covers the entire soundbox
**23**	Presence of nails to attach the soundboard to the soundbox	0: Absence 1: Presence
**24**	Presence of lacing to attach the soundboard	0: Absence 1: Presence
**25**	Presence of staples to attach the soundboard	0: Absence 1: Presence
**26**	Presence of seams to attach the soundboard	0: Absence 1: Presence
**27**	Presence of ligatures to attach the sound-board	0: Absence 1: Presence
**28**	Nails material	0: Wood 1: Metal<br>2: Wood and metal
**29**	Presence of one row of nails on one or more sides of the harp	0: Absence 1: Presence
**30**	Presence of two rows of nails on one or more sides of the harp	0: Absence 1: Presence
**31**	Lateral position of the nails	0: Absence of nails on the sides of the harp<br>1: Presence of nails on the sides of the harp
**32**	Front position of the nails	0: Absence of nails on the front of the harp<br>1: Presence of nails on the front of the harp
**33**	Dorsal position of the nails	0: Absence of nails on the back of the harp<br>1: Presence of nails on the back of the harp

N°	Character	States
**34**	Lateral position of the lacing	0: Absence of lacing on the sides of the harp<br>1: Presence of lacing on the sides of the harp
**35**	Front position of the lacing	0: Absence of lacing on the front of the harp<br>1: Presence of lacing on the front of the harp
**36**	Dorsal position of the lacing	0: Absence of lacing on the back of the harp<br>1: Presence of lacing on the back of the harp
**37**	Tight lacing	0: No lacing is tightened on the harp<br>1: All or part of the lacing is tightened
**38**	Loose lacing	0: No lacing is loosened on the harp<br>1: All or part of the lacing is loosened
**39**	Wide lacing	0: No lacing is wide on the harp<br>1 : All or part of the lacing is wide
**40**	Presence of hairs on the soundboard	0: Absence<br>1: Presence on all the soundboard<br>2: Presence except on the front of the soundboard
**41**	Presence and number of corners	0: Absence 1: Presence of 2 corners<br>2: Presence of 4 corners
**42 ***	Presence of a seal reinforcement	0: Absence 1: Presence
**43**	Presence of a suspension cord	0: Absence 1: Presence
**44**	Presence of one or more mirlitons	0: Absence 1: Presence
**45**	Soundholes location	0: Soundholes are located on the soundboard<br>1: Soundholes are located on the soundbox
**46**	Number of soundholes	0: No soundholes 1: 1 soundhole<br>2: 2 soundholes 3: 3 soundholes<br>4: 4 soundholes 5: 5 soundholes<br>6: 6 soundholes 7: 25 soundholes
**47**	Presence of a soundhole or more at top left of soundboard	0: Absence 1: Presence
**48**	Presence of a soundhole or more at top center of soundboard	0: Absence 1: Presence
**49**	Presence of a soundhole or more at top right of soundboard	0: Absence 1: Presence
**50**	Presence of a soundhole or more in the middle left of soundboard	0: Absence 1: Presence
**51**	Presence of a soundhole or more in the middle right of soundboard	0: Absence 1: Presence
**52**	Presence of a soundhole or more at bottom left of soundboard	0: Absence 1: Presence
**53**	Presence of a soundhole or more at bottom center of soundboard	0: Absence 1: Presence
**54**	Presence of a soundhole or more at bottom right of soundboard	0: Absence 1: Presence

N°	Character	States
**55**	Soundhole shape	0: Angular 1: Rounded<br>2: Greek cross<br>3: Combination of angular and rounded soundholes
**56**	String holder layout	0: Absence of a string holder<br>1: Internal string holder<br>2: External string holder<br>3: Mixed string holder
**57**	String holder attachment method	0: String holder embedded in the soundbox<br>1: String holder inserted in the soundbox<br>2: Monoxyl tailpiece with the neck
**58**	Presence of a ligature with the string holder	0: No ligature 1: Internal ligature<br>2: External ligature
**59**	Presence of holes in the string holder or in the soundboard serving as a string holder	0: Absence 1: Presence
**60**	Method of retaining strings on the string holder	0: Strings tied to or around the string holder<br>1: Strings tied to a cleat<br>2: Strings tied to themselves
**61**	Material of cleats to which ropes are attached	0 : Raw material (wood, plant, leaf, plant fiber, pearl, etc.)<br>1: Manufactured material (metal, leather, iron, fabric, etc.)
**62**	Neck morphology	0: Arched 1: Bent<br>2: S-shaped
**63**	Shape of the neck section	0: Circular 1: Angular
**64**	Presence of a fork on the neck	0: Absence 1: Presence
**65**	Method of attaching the neck to the soundbox	0: Plugged in 1: Inserted<br>2: Monoxyl<br>3: Placed on the soundbox<br>4: Placed on the soundbox and inserted
**66**	Neck straight length (in centimeters)	0: [18.3; 40.35[1: [40.35; 62.4[<br>2: [62.4; 84.45[3: [84.45; 106.5]
**67**	Neck curved length to straight length (in centimeters) ratio	0: [0.802; 0.9882875[1: [0.9882875; 1.174575[<br>2: [1.174575; 1.3608625[3: [1.3608625; 1.548]
**68**	Presence of pegs or holes in the neck	0: Absence 1: Presence
**69**	Morphology of pegs head	0: Flat 1: Rounded
**70**	Morphology of pegs knob	0: No knob 1: Choked knob<br>2: Protruding knob
**71**	Pegs material	0: Wood 1: Metal<br>2: Ivory or bone 3: Wood and rope
**72**	Peg mounting direction	0: Right to left 1: Left to right<br>2: Front to back or back to front
**73**	Peg mounting angle	0: Perpendicular 1: Angled
**74**	Presence of holes in the pegs	0: Absence 1: Presence

N°	Character	States	
**75**	Presence of a gap between the body and the head of the pegs	0: Absence	1: Presence
**76**	Presence of a notch on the pegs	0: Absence	1: Presence
**77**	Presence of a fork on the pegs	0: Absence	1: Presence
**78**	Increased peg diameter	0: Peg diameter does not increase from body to head<br>1: Peg diameter increases from body to head	
**79**	Decreased peg diameter	0: Peg diameter does not decrease from body to head<br>1: Peg diameter decreases from body to head	
**80**	Presence of a ridge on the pegs	0: Absence	1: Presence
**81**	Strings material	0: Plant fiber<br>2: Wire<br>4 : Combinations	1: Animal fiber<br>3: Nylon
**82**	Presence of twists on the strings	0: Absence<br>1: Presence on all or part of the strings	
**83**	Theoretical number of strings	0: 1 strings<br>2: 5 strings<br>4: 7 strings<br>6: 9 strings<br>8: 11 strings<br>X: 13 strings	1: 4 strings<br>3: 6 strings<br>5: 8 strings<br>7: 10 strings<br>9: 12 strings

The characters have been designed to maximize their independence, by examining on a case-by-case basis the various possible combinations and the implication of each coding procedure. For the majority of characters, Pleijel's D procedure (1995:310) was used, i.e. « treating every observable feature as an individual absent/present character. » For example, the attachment of the soundboard to the soundbox on a single harp can vary greatly, with several simultaneous modes of attachment (e.g. nails and lacing, see characters 23-27), at different locations on the soundbox (see characters 31-36) and in different ways (e.g. tightening of lacing, see characters 28-30 and 37-39). For example, the soundboard of the GR17MCODMEJE125A harp is attached only by lacing (char. 24, state 1: 24^{1}) on the sides (34^{1}) and front (35^{1}), but not on the back (36^{0}). This lacing is tight (37^{1}) in some places and loose (38^{1}) in others, but never wide (39^{0}). Similarly, the soundboard of harp SF22MGABPOGWE651A is attached by nails (23^{1}) and lacing (24^{1}). There is only one row of nails (29^{1}, 30^{0}) on the sides of the harp (31^{1}, 32^{0}, 33^{0}). The lacing is only on the back of the harp (34^{0}, 35^{0}, 36^{1}) and is wide (37^{0}, 38^{0}, 39^{1}).

Supplementary Table S3 - Data matrix

Harps are listed in the same order as in Supplementary Table S1. The states of the 83 characters for each harp are listed in the same order as in Supplementary Table S2. Harps 230, 245, 246, and 247 in this table are the outgroup harps; their rows are shaded in gray for easier identification.

N°	Harp identifier in the matrix	Character states
**1**	NC17MCODNGBMZ001A	01010100000010100120001000001010 0?????? 0001002100000011100110111011110010001000 0002
**2**	NC17MCODNGBMZ002A	121101000001141011200010000001100??????<br>20000021000000111001100010121100000010000012
**3**	NC17MCODNGBKA003A	111101000001101001300010000001010??????<br>00000020001100012001100010111100100010100002
**4**	NC17MCODNGBKA005A	1011010000000110013001010 00??????<br>001100001100200100100110111010000211101001001 00002
**5**	NC17MCODNGBKA006A	10110100010001100130010 1000??????<br>0011000011002001001001101110100001111010010000 0002
**6**	NC17MCODMANZA009A	011101000000101001200010000010100??????<br>00010020000110011001100110021100100010100002
**7**	NC17MGABFANG010A	110010000001001001200010000010110??????<br>00000031010000111001100003011110000100100005
**8**	NC17MGABFANG011A	121011000001111001200010000110101??????<br>10000021000000111001100100201100000010100307
**9**	NC17MCODBANDA012A	0001000000000110113001010 00??????<br>0011000010002001001001101110101002112010000000002
**10**	NC17MCODBANDA014A	00000000000111000140010 1000??????<br>1011000210002100000011100 10?10000211001000001000 02
**11**	NC17MCODBANDA015A	0100000000010110014001010 00??????<br>101100021000210000001110110?110002110010000010000 2
**12**	NC17MCODZANDE017A	00110000000011100140010 1000??????<br>0011000211002000011001100 1??10000211201010001000 02
**13**	NC17MCODNGBDI023A	011100000000110110000100000010100??????<br>000000200011000120011000100211000000000100002
**14**	NC17MCODNGBDI024A	111100000000111011000010000001100??????<br>00010020001000113001100010111100100000100002
**15**	NC17MCODNZKRA026A	1111000000010110014001010 00??????<br>0011000210002001001001101110000002111010000001000 2
**16**	NC17MRCANZKRA027A	0011000000001110114001010 00??????<br>1011000211002001001001100110100002111010010010000 2
**17**	NC17MCODBARBO028A	0011000000000100013001010 00??????<br>00110001100021000000111001100000011110000110100002

N°	Harp identifier in the matrix	Character states
**18**	NC17MCODBARBO029A	0001000000000110114001010000??????<br>10110002100020010010011011100000021120100100100002
**19**	NC17MCODSANGO030A	01010000000011100120001000000001110??????<br>10000010000100012001100010111100000000100002
**20**	NC17MRCASERE031A	001100000000111011300101000??????<br>00110000100021000000111011101000021110100000000002
**21**	NC17MCODALUR032A	000000000000011001100001000??????<br>1000100001002100000110??12?00000211001000000000003
**22**	NC17MUGAGANDA034A	011000100000131001300101000??????<br>100001000000100001000111 01??00011111000010010015
**23**	NC17MNGAKILBA035A	111000010001001001200101000??????<br>1011002001004000111011300 10?00001111001000101002 04
**24**	NC17MCODBUDU038A	0000000000001011012000110000101001010010001 0?<br>000000000?10111000101111001000101000 02
**25**	NC17MCODMGBTU039A	011000000000120101200010000010100??????<br>00100010000010011011??0100111110001100100002
**26**	NC17MCODMGBTU040A	000100000000011001300101000??????<br>0011000010002001001001100 1??000002112000100011000 2
**27**	NC17MCODMGBTU041A	1011000000001111001300101000??????<br>1011000010002001100001101 10?0000111120001000000112
**28**	NC17MCODMGBTU042A	100100000000011011300101000??????<br>10110000100021000000111001??0000021120100000100002
**29**	NC17MCOGNGBKA068A	1200100000002111001300101000??????<br>10101000000001000000001110011000031111001000000000 07
**30**	NC17MRCANGBKA069A	1100110000011110013001010000??????<br>101110100000100000001010010?0103111110100100100007
**31**	NC17MCMRFALI086A	0000000000010110113001010000??????<br>0011000010102000001010300 10?0000111100100001100112
**32**	GR17MCODNGBKA089A	11?1010000000001001200010000010100??????<br>00000020010010013001100010111100100000100002
**33**	GR17MGABFANG091A	00?01000000000010012000100001101 10??????<br>000000310100001010011000030111101001001 00005
**34**	GR17MGABFANG093A	11001000000100100120001000021 0110??????<br>0001003101000011100110000311111010000010 0005
**35**	GR17MGABFANG094A	01?0100000000010012000100001101 00??????<br>0001003101000010100110000301110010000010 0005
**36**	GR17MCODNGBDI096A	01110000000000110010000100001101 00??????<br>100000200100100130011000100111000000000000 00?
**37**	GR17MCODZANDE100A	01?10000000001100140110100 0??????101100?<br>21000200100100110011010001111200010001 10002
**38**	GR17MCODZANDE101A	01?100000000011001400101000??????<br>10110002110020010010011001 0?110002110000000010000 2
**39**	GR17MCODNZKRA103A	01?0000000010110014001010 00??????<br>1011000211002001001001100 11?100002110010000000000 2

N°	Harp identifier in the matrix	Character states
**40**	GR17MCODZANDE111A	00?1000000000110014001010000??????<br>0011000210002001001001101110000002112010100010002
**41**	GR17MCODMGBTU116A	00?1000000000110114001010000??????<br>10110002100020010010011001100000011120101000110002
**42**	GR17MCODALUR121A	1010000000001110011001010000??????<br>10001000000021000000111101100000121100100000100003
**43**	GR17MCODBARI122A	00?1000000000110014011010000??????001001?<br>2100021000000111001??00000211100000010110002
**44**	GR17MNGAIRGWE123A	10?0000000010110011001010000??????<br>0011002011003010001011210100?0100011100110010100204
**45**	GR17MCODMEJE125A	00?1000000000110014011010000??????110110?<br>21000210000001110011010000111100010000110002
**46**	GR17MTCDMASSA127A	111000010002111001100101000??????<br>1010100000002000001011210100?00000211001000000100312
**47**	GR17MCODSAMBA128A	00?1000000000111001301101000??????001001?<br>01000210000001110011010000211201000100000002
**48**	GR17MRCAYAKMA139A	1011000000001111001400101000??????<br>1011000210002001001001100110100011111010000011000002
**49**	GR17MCODZANDE147A	010000000000111001301101000??????001100?<br>0100020010010011001 1?00000111121000000110002
**50**	GR17MRCALINDA149A	1100000000010110014001010000??????<br>1011100210002001001001100 1??11000211001 0?000100002
**51**	GR17MRCAMBIYI151A	00?000000000010001400101000??????<br>00110002100021000000111001 1?00000211101001000000002
**52**	GR17MGABKELE159A	11?010000000002000120001000001 0100??????<br>00010010000000111001 1?00031011101001001000005
**53**	GR17MRCABANDA161A	10000000000101100140010100 0??????<br>10110002100020010010011011??10000211001000000000002
**54**	GR17MCODZANDE162A	00010000000001110013001010000??????<br>0011000011002001001001100 10?0000011110100000110002
**55**	GR17MGABTSOGO163A	000010000000100100120001001 0110100??????<br>000100100000000111001100003011110 00?00011000?
**56**	GR17MUGAGANDA164A	001000100000131001300101000??????<br>001001000000010000100011101 0?00011111100001001000015
**57**	GR17MUGAGANDA165A	111000100000131001300101000??????<br>001001000000010000100011101 0?00011111100001001000015
**58**	GR17MCODNGBKA166A	1001010000000111001300101000??????<br>1011000010002001001001100 10?010001111010010000000 2
**59**	GR17MCODNGBKA167A	11110100000001110013001010000??????<br>10110000100020010010011001 0?1100011110100000110002
**60**	GR17MCODNGBKA168A	1111010000000111011300101000??????<br>00110002100020000110011001??10000210001010001000003
**61**	GR17MCODZANDE170A	0011000000001011001400101000??????<br>00110002100020010010011011 0?00000111100001001000002

N°	Harp identifier in the matrix	Character states
**62**	GR17MRCASABGA171A	1011000000011110014001010000??????<br>10110002100021000000111001 0?110001111010010010000 2
**63**	GR17MGABFANG174A	110010000001001001200010000010100??????<br>000000310100001110010?00030211001000000000005
**64**	GR17MCODMGWDI176A	0001000000000110014001010000??????<br>1011000210002001001001101 10?100001111010010010000 2
**65**	GR17MRCABANDA177A	0001000000000110014001010001??????<br>101100021000200100100110 11??01000111101001001000 02
**66**	GR17MRCAMGBTU178A	00110000000001100140001000011 0100??????<br>021000200100100110 01??1000011110001000111002
**67**	GR18MCODZANDE180A	010100000000041001200010000110100??????<br>00010020001100013001100010111110100000000002
**68**	GR18MCODZANDE181A	110100000000121001200010000001100??????<br>000000210000001130011000101111001000000000002
**69**	GR18MCODZANDE183A	0011000000001110014001010000??????<br>00110002110020001100011011100000021110100000110002
**70**	GR18MCODZANDE184A	0001000000001110114001010000??????<br>10110002100020011000011011100000031110100000110002
**71**	GR18MCODZANDE186A	00110000000001100140010100 0??????<br>00110002110021000000111001102000021100100000100002
**72**	GR18MCODZANDE189A	00010000000001100140010100 0??????<br>00110002110020010010011011100000021100100010100002
**73**	GR18MCODNZKRA191A	00010000000001100140010100 0??????<br>10110012110020010010011011101000021110100000110002
**74**	GR18MCODZANDE192A	00110000000001101140010100 0??????<br>1011000211002001001001101 1??00000211101000001 10002
**75**	GR18MCODZANDE196A	0011000000001110014001010000??????<br>10110002100020010010011001101000021110100000110002
**76**	GR18MCODZANDE199A	00110000000001100140010100 0??????<br>10110002110020010010011001??00000211101000001 10002
**77**	GR18MCODZANDE204A	0111000000011110014001010000??????<br>10110002100020010010011011100000011110100100100002
**78**	GR18MCODNGMBE205A	0100000000011110014001010000??????<br>10110002100021000000111001101000021100100010100002
**79**	GR18MCODZANDE207A	01110000000101101140110100 0??????101101?<br>210002100000011100110000002110000000 10000002
**80**	GR18MCODZANDE208A	00110000000001100140010100 0??????<br>10110002110021000000111001100000011110100000110012
**81**	GR18MCODMGBTU227A	00010000000001100130010100 0??????<br>00110000100020010010011001100000011120001100100002
**82**	GR18MCODMGBTU228A	01010000000001100140010100 0??????<br>10110002100020010010011001100000011110000100100002
**83**	GR18MCODMGBTU230A	0101000000000110114001110001101101011000211002001 00<br>100110012?000001111000110010 0012

N°	Harp identifier in the matrix	Character states
**84**	GR18MCODMGBTU231A	1010000000011100014001010000??????<br>1011000210002100000011100 12?100001110010100000302
**85**	GR18MCODZANDE233A	111000000001111001300101000??????<br>10110010110020001100011001101000121110100000110002
**86**	GR18MCODMGBTU234A	111100000001111001400101000??????<br>10110002100020001100011001101000111110100000110002
**87**	GR18MCODMGBTU235A	111100000001111001400101000??????<br>11110002100021000000111011100000111110100000110012
**88**	GR18MCODZANDE236A	011100000000111001400101000??????<br>00110002100020010010011011100000021120100000000002
**89**	GR18MCODZANDE237A	112100000001111011400101000??????<br>1001000210002001001001101 1??0000111120100000111012
**90**	GR18MCODZANDE238A	111000000001111001400101000??????<br>10110002110020001100011001101000121110100100100012
**91**	GR18MCODMAMVU239A	111100000001111001402101000??????<br>10110002110021000000111001100000111110000010110002
**92**	GR18MCODMGBTU240A	111100000002111001400101000??????<br>10110002100020010010011001100000021110100000110002
**93**	GR18MCODMGBTU243A	000100000000111001300101000??????<br>00110001100021000000111011101000011100000000000002
**94**	GR18MCODZANDE244A	00?100000000011001400101000??????<br>1011001210002100000011100 11?10000111101000000110012
**95**	GR18MCODZANDE245A	001100000000011011400101000??????<br>0011000211002100000011101 11?0000011110100000110002
**96**	GR18MCODZANDE249A	0001000000000011001401101000??????001100?<br>2110020001100011011100000021110100100100002
**97**	GR18MCODZANDE250A	0001000000000011011400101000??????<br>1011000210002001001001100 1??00000211101001001000002
**98**	GR18MCODNZKRA251A	0001000000000011001400101000??????<br>00110002110020010010011011101000021110100100000002
**99**	GR18MCODZANDE254A	111100000000111001400101000??????<br>0011000211002000110001100 1??0000021120100000110002
**100**	GR18MCODZANDE258A	101100000001111001400101000??????<br>11110002110020001100011001102000021110100100100002
**101**	GR18MCODZANDE259A	011100000000111000140010100 0??????<br>1011000211002000110001100 12?00000111110100100100002
**102**	GR18MCODMGBTU260A	0001000000000011001300101000??????<br>10110000110020000110011001101100011110000000110002
**103**	GR18MCODMGBTU261A	0001000000000010011302101000??????<br>11110000100020001100011001101000021100000010000002
**104**	GR18MCODZANDE262A	0001000000000011011300101000??????<br>0011000011002001100001100 12?0000021110100000110012
**105**	GR18MCODZANDE265A	0001000000000011001300101000??????<br>0011000010002001001001101 12?100002111010010010 0002

N°	Harp identifier in the matrix	Character states
**106**	GR18MCODBANDA267A	0000000000000100014001010000??????<br>01110002100020010010011001100000021100000010100002
**107**	GR18MCODZANDE268A	000100000000011001300101000??????<br>10110000110020010010011001100000011120100110100002
**108**	GR18MCODMGBTU270A	000100000000011001300101000??????<br>10110000110020010010011001100000011120000100000002
**109**	GR18MCODZANDE273A	001100000000111011300101000??????<br>00110000100020010010011001101000021110100000110002
**110**	GR18MCODMGBTU275A	000100000000011001400101000??????<br>10110002100020010010011001101000011110001100100002
**111**	GR18MCODZANDE276A	000100000000011001301101000??????001100?<br>0110020010010011001101000011110100000111002
**112**	GR18MCODMGBTU283A	000100000000011011300010000010100??????<br>00010020010010011001100000021120001000100002
**113**	GR18MCODMGBTU285A	000100000000011001300101000??????<br>001100000000210000001110012?0100021110001000110002
**114**	GR18MXXXNGBDI296A	1111000000001111011301101000??????101100?<br>0100020010010011001100000021120100000100002
**115**	GR18MCODMGBTU303A	000100000000011001301101000??????<br>00110000100021000000111001100000011100100000100002
**116**	GR18MCODMGBTU304A	011100000000111001302101000??????<br>10111001100020001000111001101000021110001000110202
**117**	GR18MXXXZANDE305A	0011000000001111011300101000??????<br>10110000100020010010011001101000021110000010110002
**118**	GR18MCODMGBTU307A	000100000000111001300101000??????<br>00110000100020010010011001100000011110001000110002
**119**	GR18MCODMGBTU311A	000100000000011001301101000??????001100?<br>0100020010010011001100000011120001000110??2
**120**	GR18MCODZANDE318A	1101000000001111001300101000??????<br>10110002110021000000111001100000121120100000000012
**121**	GR18MCODNGBKA320A	0010000000001150001100101000??????<br>10110000110020010010011001101100031100100000000002
**122**	GR18MCODZANDE321A	000100000000011011300101000??????<br>00110000110020010010011001102000011120100100101002
**123**	GR18MCODMGBTU322A	000100000000011001301101000??????111100?<br>0110020001100011001101000021110001000110002
**124**	GR18MCODZANDE323A	000100000000011001300101000??????<br>00110000110021000000111011102000021110100000110002
**125**	GR18MCODMGBTU324A	000100000000011001301101000??????101100?<br>01000210000001110011001000111000000010100002
**126**	GR18MCODZANDE325A	00110000000011100131?010010010010???100?<br>0100020010010010??1100000021110100000110002
**127**	GR18MCODZANDE327A	011100000000111001300101000??????<br>10110000100021000000111001101000021110100000110002

N°	Harp identifier in the matrix	Character states
**128**	GR18MCODZANDE328A	01010000000011100130110100O??????101100?<br>0000021000000111001101000111100100010000002
**129**	GR18MCODMGBTU331A	01010000000001100130110100O??????101100?<br>0110021000000111001101100121110001000111002
**130**	GR18MCODZANDE334A	10110000000111001130010100O??????<br>101100001100200100100110011000001111200000000100002
**131**	GR18MCODNGBKA335A	11110000000111100130010100O??????<br>001110010100200011000310011011000211000000010100002
**132**	GR18MCODNGBDI336A	11110000000111100131?010000110010???????0000?<br>000000000?0??1??100002111000000000110002
**133**	GR18MCODNGBKA337A	111101000000101011200010000010100??????<br>00010020001100013001100100111100100010110002
**134**	GR18MCODNGBKA340A	0101010000000011001300101000??????<br>001100001000200100100110111010000211101000000110002
**135**	GR18MCODNGBKA341A	11110100000011101130010100O??????<br>10110000110020010010011011100000121120100100101002
**136**	GR18MCODNGBDI346A	110100000000141011300010000011100??????<br>00010020001100013001100110111100100010100002
**137**	GR18MCODNGBKA347A	01010000000012000123?010000210010???????<br>0000110000000000??1100000021100000010000?15
**138**	GR18MCODNGBKA348A	111101000000101011200010000010100??????<br>00010020001100013001100010111100100010000002
**139**	GR16MCODZANDE350A	00110000000011101140010100O??????<br>10110002110020010010011001110000031120101000100102
**140**	GR16MGABKELE351A	010010000000002000120001100011010010100100000020010<br>000111001100003111110000100000?15
**141**	GR16MRCANZKRA352A	11110000000101100140010100O??????<br>10110002110020010010011001101000011120101100001002
**142**	GR16CMRULDME353A	01000000000100100120010100O??????<br>001100200110200000101131110?01001110010?000000112
**143**	GR16MGABTSOGO356A	100011000000?20001200010000210100??????<br>00010010000000101001??0003111000100000100005
**144**	GR16MRCANZKRA358A	000100000000?1100140010100O??????<br>00110002110021000000111011??0000011110100000110412
**145**	GR16MRCAZANDE359A	000100000000?1100140010100O??????<br>001100021000200110000110011000000021000100000100012
**146**	GR16MRCANGBMA360A	221011000002?1101130010100O??????<br>1010011011001000000001110012?000311110010?000000007
**147**	GR16MGABTSOGO361A	1000100000001001001200010000111110??????<br>000100100000000111001100103111000101000100005
**148**	GR16MTCDGUIRO362A	20000000001?01001200101000??????<br>11110010010020000110011001100000111100100001000112
**149**	GR16MGABKELE363A	010010000000100100120000100O??????<br>0010010000000100000100110011?0003111110100100100005

N°	Harp identifier in the matrix	Character states
**150**	MFM18MCODMGBTU364A	000100000000011011300101000??????<br>1011000010002100000011100012?00000211200010000000002
**151**	MFM18MKENTESO366A	00000000000002000120011100010111100100110000010001<br>00011001100000011101000001000016
**152**	MFM18MGABFANG368A	0100100000010010012000100000110110??????<br>000100100000100110011001030111101001000000005
**153**	MFM18MCOGKELE374A	0100100000000200012000100000010100??????<br>0000001000000010100110010311111?00100100015
**154**	MFM18MCODGUNDA377A	0011000000001110010000100000010100??????<br>00010020001100013001100000101110100100100012
**155**	MFM18MCODBANDA381A	000000000001010001401101000??????101100?<br>210002000110001100110000001111000010010012
**156**	MFM18MCODMBUJA382A	100101000000011001400101000??????<br>10110002100021000000111011100000011120100100100002
**157**	MFM18MCODNMWSI387A	????0000000??200?123?0100001????????????0?<br>1012000000000?1??1??0000??1??000???????004
**158**	SF19MUGAGANDA393A	111000100000231001300101000??????<br>100001000000010000100013101??000111111000010010015
**159**	SF19MKENLUO394A	0010000000000200012000100000010100??????00000?<br>000000000?00011001000110221010000000301
**160**	SF19MCODZANDE404A	101100000000001100140010100????????<br>001100021000200110000011001??0000111110100000110002
**161**	SF19MCMRNTUMU406A	11001100000100100120000100????????<br>001001000000200100100010111000031111 00?00000110005
**162**	SF19MGNQFANG407A	0000100000000010012000100000210110??????<br>0000001000001001100111000301111010000??0?15
**163**	SF18TCMRNTUMU408A	1110100000011010012000100000110110??????<br>00000031010001011001 2?000311111010010 0100305
**164**	SF18TCMRNTUMU409A	0100100000000010012000100000110100??????<br>00010031010001011001100003101010100100100305
**165**	SF18TCMRNTUMU410A	0100100000010010012000100000110110??????<br>0001003101000101100110000311101010010010 0305
**166**	SF18TCMRNTUMU411A	0000100000000010012000100000110110??????<br>00010031010001011001100003001010100100100305
**167**	SF18TCMRNTUMU413A	1100100000011010012000100000110110??????<br>00010020100001031001100003111010100100100305
**168**	SF18TCMRNTUMU414A	0100100000011010012000100000110110??????<br>00010031010001011001100003011010100100100305
**169**	SF18TCMRNTUMU415A	0000100000010010012000100000110110??????<br>00010031010001031001100003011020100100100305
**170**	SF18TCMRNTUMU416A	0100100000010100120001000001101 00??????<br>0001003101000101100110000311101010010 0100305
**171**	SF18TCMRNTUMU417A	0100100000010010012000100000110100??????<br>00010031010001011001100003011010100100100305

N°	Harp identifier in the matrix	Character states
**172**	SF18TCMRNTUMU418A	1100100000010010012000100000010100??????<br>00010031010001011001100003111010100100100305
**173**	SF18TCMRNTUMU420A	0100100000010010012000100000010100??????<br>00000031010001011001100003011010100100100305
**174**	SLB19TGABTSOGO430A	0??0100000010010012000100000110100??????<br>0000001000000010100110000311110100100100305
**175**	SLB18TGABMASGO431A	0??0100000000010012000100000110100??????<br>00010010000010011001100003011110100100100305
**176**	SLB18TGABMASGO432A	0??0100000010200012000100000110100??????<br>00010010000010011001100003111110100100100305
**177**	SLB18TGABMASGO433A	1??0100000010010012000101000110100??????<br>00010010000010011001100003111110100100100305
**178**	SLB18TGABMASGO434A	0??01000000000100123?010000110100???????<br>00100100000000101001100003011010100100100304
**179**	SLB18TGABESHIR435A	1100100000011010012000100000010100??????<br>00010010000000111001100003111010100000100305
**180**	SLB18TGABNKOMI436A	1??01000000100100124?010000110100???????<br>00000100000000111001100003111010000000110305
**181**	SLB18TGABNKOMI437A	1??0100000011010?12000100000110100??????<br>0001001000000001110011?0003111010000100100305
**182**	SLB18TGABNKOMI438A	0??01000000100100123?010000110100???????<br>00000100000000111001??0003211110000100100305
**183**	SLB18TGABNKOMI439A	1??01000000110100123?010000110100???????<br>0000010000000011100 11?0003211010000100100305
**184**	SLB18TGABKNOMI440A	1??01000000110100124?010000110100???????<br>00000100000000111001100003211110000000110305
**185**	SLB18TGABBABGO441A	0??01000000?0010012000100000110100??????<br>000000100000000111001100003111110100100100305
**186**	SLB18TGABTSOGO442A	0??0100000000200012500100000110100??????<br>000000100000000111001100003111110100100100305
**187**	SLB18TGABTSOGO443A	0??01000000100100123?010000111100???????<br>00100100000000111001100003111110100100100305
**188**	SLB18TGABTSOGO444A	0??01000000110100123?010000110100???????<br>00100100000010001001100003111110000100110305
**189**	SLB18TGABPUNU445A	0??010000001001001200000100110100??????<br>000000100000000111001100003111000000000100305
**190**	SLB17TGABGALOA446A	1??01000000001?010012000100000110100??????<br>00010010000010011001100003111110100100100305
**191**	SLB17TGABNKOMI447A	1??010000002?010012000100000110100??????<br>000100100000000111001100003111110100000110305
**192**	SLB17TGABNKOMI448A	1??010000002?010?12000100000110100??????<br>000100100000000111001100003111110100100100305
**193**	SLB17TGABESHIR449A	1??010000001?010?1200010001110100??????000100?<br>00000?0??10012?0003111010100100100305

N°	Harp identifier in the matrix	Character states
**194**	SLB17TGABESHIR450A	0??010000001?01001200010000110100??????<br>200100100000001110011000032110101001001 00305
**195**	SLB18TGABMEKE451A	1??010000001001001200010000110100??????<br>000000310100010110011000031110101001001 00305
**196**	SLB18TGABMEKE452A	1??01000000010010?1200010000110100??????<br>0000003101000101100110000311101010010010 0305
**197**	SLB18TGABMEKE453A	0??010000000001001100010000110100??????<br>000000310100010110011000030110101001001 00305
**198**	SLB16TGABMASGO454A	???010000?0112000123?010000110100???????<br>0010010000000101001 1?000311111010010010 0305
**199**	SLB167GABMEKE462A	???010000?0100100120001000011 0110??????<br>000000100000010110011?0003111010100100100305
**200**	SLB16TGABMASGO463A	???010000?01001001200010000110100??????<br>10010010010000001100 11?00032111101001001 00305
**201**	SLB16TGABMEKE464A	???010000?01001001100010000110100??????<br>00010031010001011001 1?0003111010100100100305
**202**	SLB16TGABMEKE465A	???010000?01001001200010000110100??????<br>000000310100010110011?0003111??010???????305
**203**	SLB16TGABMEKE466A	???010000?02101001100010000110100??????<br>000100310100010110011?0003211??010???????309
**204**	SLB16TGABMEKE467A	???010000?01101001100010000110100??????<br>000000310100010110011?0003111010100100100305
**205**	SLB16TGABMEKE468A	???010000?01101001200010000110100??????<br>000000310100010110011?0003111010100100100305
**206**	SLB16TGABMEKE471A	???010000?01001001200010000110100??????<br>000000310100010110011?0003111010100100100305
**207**	SLB16TGABTSOGO473A	???010000?01101001200010000110100??????000100??0??<br>0?0??10011?0103111110100100100305
**208**	SLB16TGABNKOMI477A	???010000?01001001200010000110100??????<br>2001001000000001110011?000311111010000011 0305
**209**	SLB16TGABORNGU478A	???010000?01001001200010000110110??????<br>000000100000001110011?0003111110100100100305
**210**	SLB16TGABORNGU479A	???010000?01101001200010000110100??????<br>000100100000001110011?0003111110100100100305
**211**	SLB16TGABORNGU480A	???010000?0210100124?010000110100???????<br>001001000000001110011?0003211110100100100305
**212**	SLB16TGABMEKE481A	???0100000001010011000100001 10100??????<br>000100310100010310011?0003011010100100100305
**213**	SLBXXTGABMEKE482A	???01000000??01001200010000110100??????<br>000000510100010110011000 03??1010100100100305
**214**	SLBXXTGABMASGO484A	???01000000??01001200010000110100??????<br>0000001000001001100 1??0003??1110100100100305
**215**	SLB19TGABFANG488A	???0100000011010012000100001 10100??????<br>000000201000010110011000031110101001001 00305

N°	Harp identifier in the matrix	Character states
**216**	SLB19TGABFANG490A	???01000000110100120001000011010??????<br>00010020100001011001100003111020100100100305
**217**	SLB19TGABFANG495A	???01000000110100120001000011010??????<br>0001003101000101100 1??0003111110100100100305
**218**	SLB19TGABFANG496A	???01000000110100120001000011010??????000?<br>0031010001011001??000311101010010010 0305
**219**	LM20MGABTSOGO506A	???01000000000100120001000011011??????<br>20000010000000101001??0003111110100100100305
**220**	SS20MGABTSOGO483C	1100100000010010012000100001111 00??????<br>000100100000001110011100031111101000001 10305
**221**	SS20MGABPUN504B	11001000000100100123?01000011010 0???????<br>00000100000001010011001031111101001001 00305
**222**	SLB22MGABMASGO515A	0000100000000200012000100000011 00??????<br>000100200100100110011001030111101000001 10303
**223**	SS22MSSDACH518A	1310000000011200011001010 00??????<br>10000110000020010010011101100000311010000100100317
**224**	SS22MSSDACH521A	010000000000020001100101000??????<br>10001000000020010010001101100000111020000000100315
**225**	SS22MNGADUNG522A	01010100000000101110000001 0????????????<br>00110020001100002101100000001110010000 0100113
**226**	SS22MCODMGBTU525A	0101000000000110014001010 00??????<br>1011000210002001001001100110100001111000100 0110002
**227**	SS22MNGABEROM533A	110000000001111001100101000??????<br>001100001000301000101121110?01000111001100101 00203
**228**	SS22MAFGXXX535A	010000000001050001400001000??????<br>101001020100711100111122011101 01??1?03000000000301
**229**	SS22MSDNBEDA536A	000100000000011001400101000??????<br>0011000211002001001001 1001??11000111101000000110112
**230**	SS22MLBRKROU539A	01?00000010?31100????????????????????????000?<br>000000000?20000?0000010?????????????003
**231**	SS22MLBRKROU540A	???00000001??31100????????????????????????000?<br>000000000?20000?0003??0?????????????404
**232**	SLB22MSDNNUBA542A	010000000001111001400101000??????<br>001010221100210000001110011010001111001000 10000112
**233**	SLB22MRCANZKRA543A	000100000000011001400101000??????<br>101100021000200100100110 01??000001111010000011 1002
**234**	SLB22MUGATWA544A	000010000000050001401010000110010??????<br>0000002100000001110011000000011000000001 0100012
**235**	SLB22MSSDZANDE547A	000100000000011001400101000??????<br>101100021100200100100110 01??1000011120100000110202
**236**	SLB22MUGATWA548A	000010000000150001301010000110010??????<br>000000210000000111001100000001100000000 10100?12
**237**	SLB22MSSDZANDE549A	101100000001111001400101000??????<br>0011000211002001001001100110000000112010000011 0002

N°	Harp identifier in the matrix	Character states
**238**	SLB22MUGAGANDA550A	1010001000013310013001010000??????<br>00100100000010000100011101??0001111110000100100015
**239**	SLB22MUGAITESO552A	100000000001011001100101000??????<br>1000100001001000010001311110000101110000000000013
**240**	SLB22MUGAACH557A	001000000000211011300101000??????<br>1000011001002001001001110122?0000011100000110100012
**241**	SLB22MGABNKOMI559A	010010000000020001200010000110100??????<br>00000010000000111001100103011110100000110005
**242**	SS22MGABNKOMI574A	021010000001101001200010000010100??????<br>20010010000000111001100003111000100000110315
**243**	SS22MMMRXXX583A	000010000000100100121?010001110010???????<br>0000061011110110??10?0001011100100000100013
**244**	SS22MAFGXXX588A	000000000001050001400001000??????<br>001001020000711110111122011100001??1?03100000000?01
**245**	SS22MRUSXXX589A	11000000000212000111?010000110010???????<br>0000041010010120??10?2102011000000000100206
**246**	RJ22MGABTSOGO590A	01001000000100100123?010000111110???????<br>001001000000001110011?0003111110100100100305
**247**	RJ22MGABTSOGO591A	110010000001001001200010000110100??????<br>2001001000000001110011?0003111110100100100305
**248**	RJ22MUGAALUR592A	111000000000120001100101000??????<br>101110100100210000001111011?0000111010000100100307
**249**	RJ22MUGATESO596A	201000000001111001100101000??????<br>10010020010010000100011101??0001211100000000100013
**250**	RJ22MUGAACHO597A	011000000000111001200101000??????<br>10001010000021000000111101100000111100000000100114
**251**	RJ22MUGAKONJ598A	110000000000041001201101000??????<br>10110000010021000000111001100000111110000000100015
**252**	RJ22MUGAKONJ599A	000000000000120001200101000??????<br>10010000000021000100011001100000111100000000100115
**253**	RJ22MUGASOGA601A	122000000001111001300101000??????<br>100001200100100100000111011?0101121120000000110115
**254**	RJ22MUGAMADI602A	101000000000111001100101000??????<br>100010100100210000001111010?0000111110000100100014
**255**	RJ22MUGAKONJ603A	000000000000041001201101000??????<br>10010000000021000000111001100000111110000100100015
**256**	RJ22MUGALANI604A	0020000000020031001300001000??????<br>10001000000020010010011001100000011110000100100012
**257**	RJ22TUGAGANDA605A	111000100000131001300101000??????<br>10000100000010000100011101100001111110000100100105
**258**	RJ22TUGAACHO606A	121000000000120001200101000??????<br>1000101001001000100001100110000021100000000010030X
**259**	RJ22TUGAACHO607A	3330000000003350001200101000??????<br>10001010000010000100011001100000311001001000100317

N°	Harp identifier in the matrix	Character states
**260**	RJ22TUGAACHO608A	0100000000000500012001010000??????<br>10001010000020010010011001100000111001001000100309
**261**	RJ22TUGAACHO609A	32100000000022200012001010000??????<br>10001010010020010010011001100000311000000000010031X
**262**	RJ22TUGAACHO610A	22200000000022200012001010000??????<br>10001010010020010010011001100100311000000000010031X
**263**	RJ22TUGAACHO611A	1100000000001100012001010000??????<br>10001010010010001000011001100000211000000000100309
**264**	RJ22TUGAACHO612A	0100000000000200012001010000??????<br>10000110000021000000111001100000111020000100100309
**265**	RJ22TUGAACHO613A	1100000000000400012001010000??????<br>10001010000021000000111001100000111020000100100308
**266**	RJ22TUGAACHO614A	0100000000000400012001010000??????<br>10000110000021000000111001100000111020000100100309
**267**	RJ22TUGAACHO615A	1100000000001200012001010000??????<br>10001010000021000000111001100000101020000100100309
**268**	RJ22TUGALANG616A	11000000000010100123?010000110110???????<br>0000021000100000??110000021102100100010030X
**269**	RJ22TUGALANG617A	333000000000332000120000100 0??????<br>10001010000021000000111001100000311021001000100304
**270**	RJ22TUGALANG618A	1130000000012010012 3?010000110110???????<br>00000200110000000??1100000111021001000100309
**271**	RJ22TUGALANG619A	0010000000001010012 3?0100001??110???????<br>000002100000010 0??110000000110210010001003 0X
**272**	RJ22TUGATESO620A	0110000000001200012 3?010000110010???????<br>0000010000010011001110100111021001000100307
**273**	RJ22TUGATESO621A	1210000000011200012 3?010000110010???????<br>0000020010010011001110000211021001000100308
**274**	RJ22TUGAGWER622A	211000000000111100110010100 0??????<br>10001020010010000100011001100000101100000000100013
**275**	RJ22TUGAGWER623A	111000000000111100110010100 0??????<br>100010200100100100000110011000001111000000000110013
**276**	RJ22TUGASOGA624A	12200000000012500013001010000??????<br>100010100000100001000111011000002111200000000110115
**277**	RJ22TUGAGANDA595B	1110001000002310013001010000??????<br>100010200000100000001111011000011111100001001001 15
**278**	SF22MGABTSOGO633A	10101100000000200012000100000001100??????<br>000000100000000111001100003011110101100100305
**279**	SF22MGABTSOGO645A	01001000000010010012000100000110100??????<br>0000001000000001110011000 03??1110100100100305
**280**	SF22MAGOOVIM647A	13210000000000400013000100002101 00??????00000?<br>000000000?2??10?0000230????????????010
**281**	SF22MCMRTUPRI648A	1001010000010110013001010000??????<br>0011000010005001111011300 10?0000011100?00000000012

N°	Harp identifier in the matrix	Character states
**282**	SF22MGABPOGWE650A	11001000000101100130011100011010000100100010010000<br>0001110011000031111101001001000015
**283**	SF22MGABPOGWE651A	01001000000000100120001100001010000100100010010000<br>0001010012?000301111010010010000005
**284**	SF22MCODNZKRA653A	00110000100011101131?010010010010???????<br>00000200100100 10??1??201001111000000001100 02
**285**	SF22MCODZANDE658A	011100000000011001400101000??????<br>00110002110020010010011001??100001110010100010001 2
**286**	SF22MGABFANG665A	0100100000000010012000100000 10100??????<br>0000002000100013100 12?000311111010010010 0?15
**287**	SF22MTZAHA667A	1100000000010010012001010 00??????<br>10110000010020010010001001101010011100100010000102
**288**	SF22MUGAKONJ668A	1010000000000111001200101000??????<br>100010000000200011000110011?000011111000010000001 5
**289**	SS23MRCANGBMA673A	1300110001021110013001100001101 01??????<br>100000100000001010011001032111001000101 00307
**290**	SLB21TGABNZAM675A	???0100000011010?1200010000????????????<br>00000031010000101001100000111010100100100305
**291**	SLB21TGABNZAM676A	???0100000011010012000100001 10100??????<br>00010031010001011001100003111010100100100305
**292**	SLB21TGABMEKE458B	???01000000210100110001000011 0100??????<br>00000031010001011001100003111010100100100305
**293**	SLB21TGABBETSI459B	???01000000110100120001000011 0100??????<br>00000020100001011001100003111010100100100309
**294**	SLB21TGABFANG677A	???010000001001001200000100110100??????<br>00000031010001011001100003111010100100100305
**295**	SLB22TGABMEKE678A	110010000001101001200010000110100??????<br>00000031010001011001100003111010100100100305
**296**	SLB22TGABMEKE679A	111010000001101001200010000110100??????<br>00000031010001031001100003111010100100100305
**297**	SLB22TGABMEKE457B	110010000001001001200010000110100??????<br>00000031010001031001100003111010100100101305
**298**	SLB22TGABAKELE683A	0100100000000010012 3?010000110100???????<br>000001000000011100110000311111010010010 0305
**299**	SLB22TGABTSOGO684A	110010000001001001200010000110100??????<br>000100??????????1001100003111110100100100305
**300**	SLB22TGABGALOA685A	110010000001101001200010000110110??????00000?<br>000000000?1001100104111110100100100305
**301**	SLB22TGABFANG686A	110011000001101001 24?0100001 10100???????<br>0000010000010011001100003111000100000100305
**302**	SLB22TGABTSOGO687A	110010000001001001 23?0100001??100???????<br>0010010000000101001100003111110100000110305
**303**	SLB22TGABTSOGO688A	010010000001001001 23?0100001??100???????<br>0010010000000111001100003011110100000110305

N°	Harp identifier in the matrix	Character states
**304**	SLB22TGABGALOA689A	1100100000001001001200010000110100??????<br>00010010000000111001100003111110100000110305
**305**	SLB22TGABGALOA690A	0100100000000001001200000100110100??????<br>20010010000000101001100003011110100100100305
**306**	SLB22TGABTSOGO691A	111010000000101001200000100?10100??????<br>00010010000000111001100003011010200100100305
**307**	SLB22TGABFANG692A	11001000000102000123?010000110100???????<br>0010031010000111001100003111010100100100305
**308**	SLB22TGABFANG469B	1100100000001001001200010000110100??????<br>00000031010001031001100003111010100100100305
**309**	SLB22TGABFANG470B	1100100000001001001200010000110100??????<br>20000031010001001001100003111010100000110305
**310**	SLB22TGABFANG472B	0100100000001001001200010000110100??????<br>00000031010001011001100003111010100100100305
**311**	SS23MCMRNTUMU693A	0100100000001001001200010000110110??????<br>00000031010001011001100003111010100100100305
**312**	SS23MGABMASGO390B	1100100000001001001200010000110100??????<br>00000010000000101001100103111110100100100305
**313**	SS23MGABFANG391B	1100100000001001001200010000110100??????<br>00000031010001011001100003111010100100100305
**314**	SS23MGABTSOGO389B	1200100000001101001200010000110100??????<br>20000010000000111001100003211110100100100305
**315**	SS23MRCAAKA696A	010000000030001000???????????????????????<br>000030001010010??1101110011100?10010100304
**316**	SS23MRCAAKA697A	010000000030001000???????????????????????<br>000030010100110??1101110111100100010000302
**317**	SS23MRCAAKA700A	10000000000101000123?010000110110???????<br>0000010000001010??1100110111100000010000303
**318**	SS23TCMRNTUMU412B	??1010000002201001200010000110100??????<br>00000031010001011001100003??1110100100100305

Supplementary Table S4 - Character data on the majority-rule (50%) consensus cladogram

Character	Minimum number of steps	Number of steps on the cladogram	Maximum number of steps	Consistency index (CI)	Retention index (RI)
**1**	3	51	125	0.059	0.607
**2**	3	60	132	0.050	0.558
**3**	3	44	103	0.068	0.590
**4**	1	15	122	0.067	0.884
**5**	1	6	118	0.167	0.957
**6**	1	17	25	0.059	0.333
**7**	1	1	7	1.000	1.000
**8**	1	2	2	0.500	0.000
**9**	1	1	1	1.000	0/0
**10**	1	2	2	0.500	0.000
**11**	3	3	5	1.000	1.000
**12**	3	44	152	0.068	0.725
**13**	3	51	136	0.059	0.639
**14**	5	35	172	0.143	0.820
**15**	1	19	51	0.053	0.640
**16**	1	3	4	0.333	0.333
**17**	1	28	36	0.036	0.229
**18**	1	2	4	0.500	0.667
**19**	4	38	168	0.105	0.793
**20**	4	14	30	0.286	0.615
**21**	2	19	23	0.105	0.190
**22**	1	12	152	0.083	0.927
**23**	1	13	144	0.077	0.916
**24**	1	13	143	0.077	0.915
**25**	1	3	5	0.333	0.500
**26**	1	4	4	0.250	0.000
**27**	1	3	3	0.333	0.000
**28**	2	16	36	0.125	0.588
**29**	1	4	8	0.250	0.571
**30**	1	8	13	0.125	0.417
**31**	1	5	11	0.200	0.600
**32**	1	18	36	0.056	0.514
**33**	1	2	3	0.500	0.500
**34**	1	29	60	0.034	0.525
**35**	1	6	7	0.167	0.167
**36**	1	8	34	0.125	0.788
**37**	1	11	47	0.091	0.783

Character	Minimum number of steps	Number of steps on the cladogram	Maximum number of steps	Consistency index (CI)	Retention index (RI)
**38**	1	15	30	0.067	0.517
**39**	1	10	24	0.100	0.609
**40**	2	27	47	0.074	0.444
**41**	2	13	76	0.154	0.851
**42**	1	11	119	0.091	0.915
**43**	1	61	134	0.016	0.549
**44**	1	1	2	1.000	1.000
**45**	1	2	2	0.500	0.000
**46**	7	28	141	0.250	0.843
**47**	1	17	99	0.059	0.837
**48**	1	3	8	0.333	0.714
**49**	1	24	135	0.042	0.828
**50**	1	20	37	0.050	0.472
**51**	1	22	45	0.045	0.523
**52**	1	26	106	0.038	0.762
**53**	1	4	43	0.250	0.929
**54**	1	24	123	0.042	0.811
**55**	3	27	36	0.111	0.273
**56**	3	21	41	0.143	0.526
**57**	2	10	25	0.200	0.652
**58**	1	24	38	0.042	0.378
**59**	1	1	2	1.000	1.000
**60**	2	28	41	0.071	0.333
**61**	1	5	7	0.200	0.333
**62**	2	30	63	0.067	0.541
**63**	1	31	43	0.032	0.286
**64**	1	8	20	0.125	0.632
**65**	4	13	128	0.308	0.927
**66**	3	45	163	0.067	0.738
**67**	3	38	81	0.079	0.551
**68**	1	1	3	1.000	1.000
**69**	1	16	75	0.063	0.797
**70**	2	57	130	0.035	0.570
**71**	3	7	13	0.429	0.600
**72**	2	36	101	0.056	0.657
**73**	1	2	3	0.500	0.500
**74**	1	22	37	0.045	0.417
**75**	1	38	136	0.026	0.726
**76**	1	25	36	0.040	0.314
**77**	1	3	3	0.333	0.000

Character	Minimum number of steps	Number of steps on the cladogram	Maximum number of steps	Consistency index (CI)	Retention index (RI)
**78**	1	35	48	0.029	0.277
**79**	1	28	70	0.036	0.609
**80**	1	9	9	0.111	0.000
**81**	4	32	144	0.125	0.800
**82**	1	35	60	0.029	0.424
**83**	10	36	172	0.278	0.840
